# Next generation of tumor-activating type I IFN enhances anti-tumor immune responses to overcome therapy resistance

**DOI:** 10.1038/s41467-021-26112-2

**Published:** 2021-10-07

**Authors:** Xuezhi Cao, Yong Liang, Zhenxiang Hu, Huiyu Li, Jiaming Yang, Eric J. Hsu, Jiankun Zhu, Jin Zhou, Yang-Xin Fu

**Affiliations:** 1 Department of Pathology, UT Southwestern Medical Center, Dallas, TX 75390 USA; 2 LivzonBio, Inc., Zhuhai, Guangdong 519045 China

**Keywords:** Cancer microenvironment, Tumour immunology, Tumour immunology

## Abstract

Type I interferon is promising in treating different kinds of tumors, but has been limited by its toxicity, lack of tumor targeting, and very short half-life. To target tumors, reduce systemic toxicity, and increase half-life, here we engineer a masked type I IFN-Fc (ProIFN) with its natural receptor connected by a cleavable linker that can be targeted by tumor-associated proteases. ProIFN has a prolonged serum half-life and shows an improved tumor-targeting effect. Interestingly, ProIFN-treated mice show enhanced DC cross-priming and significant increased CD8^+^ infiltration and effector function in the tumor microenvironment. ProIFN is able to improve checkpoint blockade efficacy in established tumors, as well as radiation efficacy for both primary and metastatic tumors. ProIFN exhibits superior long-term pharmacokinetics with minimal toxicity in monkeys. Therefore, this study demonstrates an effective tumor-activating IFN that can increase targeted immunity against primary tumor or metastasis and reduce periphery toxicity to the host.

## Abstract (web summary)

Several studies have demonstrated that IFN-I administration can boost anti-tumor immune response against solid tumors. Here, to overcome the limitations of systemic IFN-I therapy (side effects, short half-life), the authors design a masked IFN-I prodrug activatable by tumor-associated proteases, showing preserved anti-tumor activity but reduced toxicity.

## Introduction

Interferons (IFNs) belong to the large class of proteins known as cytokines, which are used for communication between cells to trigger the protective defenses of the immune system that help eradicate pathogens^1^. There are three classes of IFNs: type I, type II, and type III^2^. Type I IFNs (IFN-Is), all of which have considerable structural homology, include IFN-α which can be further subdivided into different subtypes^3^. All IFN-Is bind to a specific cell surface receptor complex known as the IFN-α/β receptor (IFNAR)^4^. The IFN-I receptor is composed of two subunits, IFNAR1 and IFNAR2, which are associated with the Janus activated kinases (JAKs) tyrosine kinase 2 (TYK2) and JAK1, respectively^5,6^. Activation of the JAKs that are associated with the IFN-I receptor results in induction of many IFN stimulated genes (ISGs)^7^. IFN-Is contribute to activation and maturation of dendritic cells (DCs), leading to better antigen processing and presentation for T cell priming and re-activation^8^. For anti-tumor effects, IFN-Is can also inhibit proliferation, control angiogenesis, modulate apoptosis, differentiation, migration, and cell surface antigen expression^9,10^.

For solid tumor treatment, IFN-α2b is an effective adjuvant therapy for melanoma patients at high risk of recurrence that has been approved by regulatory authorities worldwide^11^. Intriguingly, in human metastatic melanoma, there was a positive correlation between an IFN-I transcriptional profile and T cell markers in metastatic tumor tissue^12^. Two independent studies have demonstrated that endogenous IFN-I signaling is critical for the innate immune recognition of a growing tumor through signaling on CD8α^+^ DCs^12,13^. Several studies revealed exogenous administration of IFN-I can boost the innate and adaptive immune responses against solid tumors. Furthermore, adenoviral-mediated intratumoral expression of IFN-I is sufficient to selectively expand antigen-specific T cells leading to complete tumor regression^14^. An armed therapeutic antibody with IFN-I is more potent than the first generation of antibody for controlling antibody-resistant tumors by increasing direct killing of lymphomas^15^ or bridging suppressed innate and adaptive immunity in the tumor microenvironment^16^. However, the antitumor effect of systemic IFN-I therapy is often accompanied by severe side effects, including inflammatory symptoms and direct tissue toxicity^17^. IFN toxicities and frequent use due to its short half-life increase the risk of poor treatment compliance, fail to reach the therapeutic window^11^. One solution to these problems is the conjugation of the polymer polyethylene glycol (PEG) to IFN, which significantly increases the in vivo half-life and avoids frequent administration^18,19^. However, there are no overall significant differences between the incidence of adverse effects (AEs) experienced by patients comparing Peg-IFNα-2b and IFNα-2b, as well as Peg-IFNα-2a and IFNα-2a^20^. Therefore, Peg-IFN has been approved to increase half-life but still lacks the tumor-targeting capabilities. It is also challenging to manufacture desired Peg-IFNα-2b with precise numbers and positions of PEGylation.

In this work, we develop the next generation of IFN by engineering and masking IFN-I into a prodrug, denoted as ProIFN. We mask IFN-I with one subunit of its natural receptor to reduce peripheral activity; It contains a human IgG1 Fc fragment for prolonged half-life and easy manufacture and a cleavable linker for reversibly controlling its activity through tumor-associated proteases cutting. With this design, we generate the next generation of IFN-I with significantly reduced toxicity, prolonged half-life, and tumor-activating property. This safe and effective ProIFN can address numerous clinically relevant scenarios, including advanced cancer resistance to immune checkpoint blockade or radiation therapy in both primary and metastatic tumors. Taken together, we provide potent immunotherapy that can safely combat many difficulties to treat cancers.

## Results

### Masked type I IFN prodrug with interferon-alpha/beta receptor can block IFN activity and reactivate in vitro

**Fig. 1:**
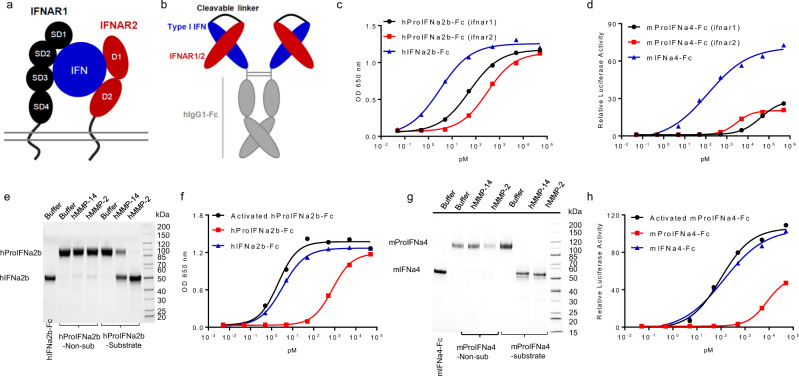
Blockage of IFN-I activity by IFNAR1/2. **a** Schematic depicting IFN-I binds to IFNAR1 and IFNAR2. **b** Design of IFN-I prodrug which IFNAR1/2 extracellular domain fused to IFN-Fc via a cleavable linker. **c** IFN activity of human IFNAR1/2 masked IFNa2b-Fc was assessed using a human IFN reporter cell line (*n* = 2). **d** IFN activity of mouse IFNAR1/2 masked IFNa4-Fc was assessed using mouse IFN reporter cell line (*n* = 2). **e** Reducing SDS-PAGE analysis of hProIFNa2b-Fc as well as MMP-14- or MMP-2-cleaved hProIFNa2b-Fc. The experiment was repeated two times independently with similar results. **f** IFN activity of MMP-activated hProIFNa2b-Fc, hIFNa2b-Fc, and hProIFNa2b-Fc was assessed via human IFN reporter cell line (*n* = 2). **g** Reducing SDS-PAGE analysis of mProIFNa4-Fc as well as MMP-14- or MMP-2-cleaved mProIFNa4-Fc. The experiment was repeated two times independently with similar results. **h** IFN activity of MMP-activated mProIFNa4-Fc, hIFNa2b-Fc, and mProIFNa4-Fc was assessed via mouse IFN reporter cell line (*n* = 2). Human IFN activity was assessed by 293T-Dual™ hSTING-R232 cells via secreted embryonic alkaline phosphatase activity. Mouse IFN activity was assessed by RAW-Lucia ISG cells via secreted coelenterazine-utilizing luciferase activity. Source data are provided as a Source Data file.

The interferon-α/β receptor (IFNAR) is a heterodimeric cell surface receptor composed of two subunits: the low affinity subunit, IFNAR1, and the high affinity subunit, IFNAR2 (Fig. 1a). ProIFN for both human IFNa2b and mouse IFNa4 were generated, as human type I IFN has no cross-activity in mice. The N-terminal ligand binding domain of either IFNAR1 or IFNAR2 was genetically fused to the N-terminal of IFN-I through a protease-cleavable linker. We have tested a wide range of protease-cleavable linkers (such as SGQLLGFLTA^21^). They are substrate sequences for matrix metallopeptidases (MMPs), such as MMP-14 and MMP-2, that are enriched in a variety of cancers^22^. The TCGA (The Cancer Genome Atlas) data also show the increased level of MMP-14 and MMP-2 in most kinds of tumor tissues (Supplementary Fig. 1). To increase the half-life of the fusion protein, the Fc domain of human IgG1 was linked to the C-terminus of the construct (Fig. 1b). Masked human IFNa2b with IFNAR1 or IFNAR2 decreased IFN activity by 18- and 99-fold, respectively (Fig. 1c). Both IFNAR1- and IFNAR2-masked mouse IFNa4 decreased IFN activity by over 1000-fold (Fig. 1d). These results suggest that the IFNAR domain can effectively block IFN-I’s activity. Because IFNAR2 blocked more effectively than IFNAR1 for human IFN, we used and focused on the IFNAR2-masked IFN-I (hProIFNa2b-Fc for human and mProIFNa4-Fc for mouse) in the following studies.

To investigate whether cleavage of ProIFN could restore IFN activity, hProIFNa2b-Fc was incubated with purified MMPs such as human MMP-14 or human MMP-2. Site-specific removal of the mask upon cleavage was confirmed by SDS-PAGE (Fig. 1e). We demonstrated that cleavage of hProIFNa2b-Fc by either MMP-2 or MMP-14 was efficient. In contrast, ProIFN with a control linker that cannot be cleaved (Non-sub) was resistant to both MMP-14 and MMP-2 cleavage. IFN activity of fully precleaved hProIFNa2b-Fc was restored to that of unmasked hIFNa2b-Fc (Fig. 1f). Similarly, mProIFNa4-Fc was incubated with purified human MMP-14 or MMP-2, resulting in near complete site-specific removal of the mask by either protease, which was confirmed by SDS-PAGE (Fig. 1g). IFN activity of precleaved mProIFNa4-Fc was also restored to that of unmasked mIFNa4-Fc (Fig. 1h). Therefore, ProIFN showed greatly decreased activity while completely restored its activity after MMP treatment.

### Reduction of toxicity by ProIFN

**Fig. 2:**
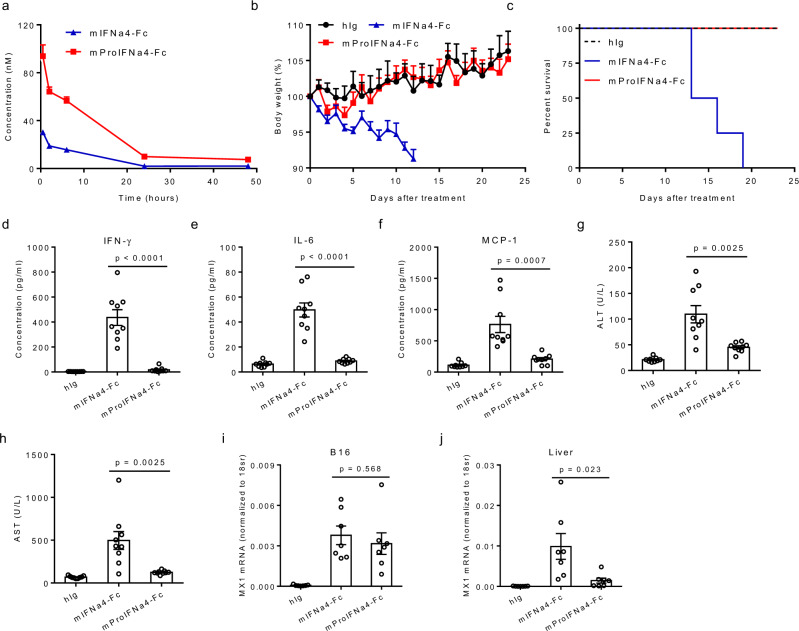
In vivo assessment of toxicity. **a** The pharmacokinetics of mProIFNa4-Fc and mIFNa4-Fc were compared in C57BL/6 mice following one intravenous (i.v.) dose at 1 nmol (*n* = 4 animals). **b**, **c** Healthy C57BL/6 J mice were intraperitoneally (i.p.) treated with 1 nmol of hIg, mIFNa4-Fc, or mProIFNa4-Fc, every 3 days for 6 times (*n* = 4 animals). Body weight (**b**) and survival curve (**c**) were shown. **d**–**h** Healthy C57BL/6 J mice were intraperitoneally (i.p.) treated with 1 nmol of hIg, mIFNa4-Fc, or mProIFNa4-Fc, every 3 days for three times (*n* = 9 animals). Plasma samples were collected 2 days after the last treatment. The levels of IFN-γ (**d**), IL-6 (**e**) and MCP-1 (**f**) were quantified by cytometric bead array (CBA). The levels of ALT (**g**), AST (**h**) were determined by UTSW Metabolic Phenotyping Core. **i**, **j** C57BL/6 J mice were s.c. inoculated with 5 × 10^5^ B16 cells and i.p. treated with 1 nmol of hIg, mIFNa4-Fc, or mProIFNa4-Fc on day 11 (*n* = 7 animals). Liver and tumor tissues were harvested 48 h after treatment. Intracellular RNA was extracted for RT-qPCR assay to determine expression levels of MX1 in B16 tumors (**i**) and livers (**j**). Data are reported as mean ± s.e.m. Two-tailed *t*-tests were performed to calculate *p*-values. Source data are provided as a Source Data file.

To evaluate ProIFN in vivo, we first compared the pharmacokinetics of mProIFNa4-Fc with unmasked mIFNa4-Fc in C57BL/6 mice. Following intravenous administration at a dose of 1 nmol, mProIFNa4-Fc showed a longer half-life to that of mIFNa4-Fc, which were 6.332 and 4.757 h, respectively. Furthermore, mProIFNa4-Fc also displayed a much higher serum concentration than mIFNa4-Fc, suggesting a higher amount of drug exposure of mProIFNa4-Fc in plasma (Fig. 2a). We next tested the consequences of the mask on toxicity in healthy mice. C57BL/6 J mice treated with multiple doses of mIFNa4-Fc steadily lost weight, died, or declined in health to the point of requiring euthanasia, whereas minimal weight decline was observed with mProIFNa4-Fc treatment (Fig. 2b–c). The increased weight loss in mIFNa4-Fc treated mice occurred after the fourth treatment, indicating that three injections of either IFN construct are nonlethal and can be used to observe tumor growth curve for a few weeks. As a result, we used this dose and frequency in the following studies.

Next, we probed the consequences of the mask on the production of inflammatory cytokines such as interferon- γ (IFN-γ), interleukin-6 (IL-6), and monocyte chemoattractant protein-1 (MCP-1). In contrast to mProIFNa4-Fc, mIFNa4-Fc elicited a much higher level of inflammatory cytokines two days after the last treatment (Fig. 2d–f). To further demonstrate the safety of ProIFN, we tested the levels of functional parameters associated with liver and kidney. For liver function, unmasked mIFNa4-Fc induced much higher production of ALT and AST than mProIFNa4-Fc in the blood of treated mice (Fig. 2g–h). We hypothesized that the reduced liver toxicity of ProIFN is due to the lower IFN activity in the liver. Indeed, in B16 tumor-bearing mice, mProIFNa4-Fc induced a much lower level of an interferon-stimulated gene (ISG) MX1 than unmasked mIFNa4-Fc, while both induced similar MX1 expression levels in B16 tumor tissues (Fig. 2i–j). For kidney function, we tested sodium, potassium, calcium, urea, inorganic phosphorus, and uric acid. mIFNa4-Fc treatment showed slightly increased potassium level and decreased uric acid level. mProIFNa4-Fc treatment did not show any significant change of kidney functional parameters (Supplementary Fig. 4). These results demonstrate that the masked ProIFN has reduced toxicity and can prevent the host from systemic treatment inducing cytokine storm and severe liver toxicity.

### ProIFN preserves potent anti-tumor activity in vivo

To explore the anti-tumor effect of ProIFN, we first determined the expression levels of tumor-enriched enzymes such as MMP-2, MMP-9, MMP-11, MMP-14, fibroblast activation protein alpha (FAP), and urokinase plasminogen activator (uPA) in a variety of tumor lines. MC38 (colon cancer), B16 (melanoma), and LLC (lung cancer) all expressed high MMP-14. High MMP-2 expression was found in MC38 and high MMP-9 was found in LLC (Supplementary Fig. 2). Meanwhile, low expression levels of tissue inhibitors of metalloproteinases (TIMP-3 and TIMP-4) were found in all three tumor lines (Supplementary Fig. 3). These data suggest that MMP-2, MMP-9, and MMP-14 can be practical tumor-enriched enzymes for ProIFN activation in vivo. Since the substrate chosen can be cleaved by MMP-14 or MMP-2, all three tumor lines appeared to be feasible for our studies.

**Fig. 3:**
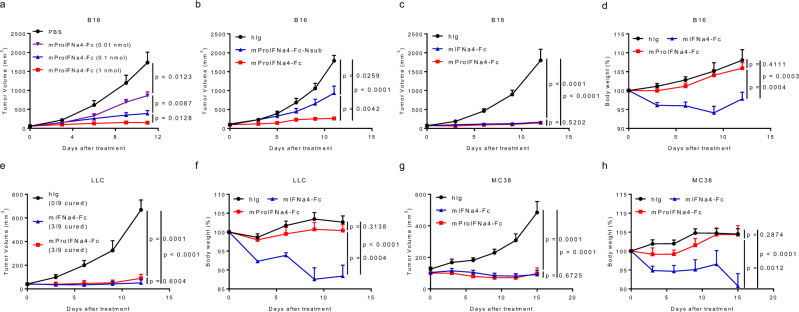
In vivo assessment of efficacy. **a** Dose-dependent anti-tumor effect of mProIFNa4-Fc. C57BL/6 J mice were s.c. inoculated with 5 × 10^5^ B16 tumor cells and i.p. treated with 0, 0.01, 0.1, or 1 nmol of fusion protein on day 7, 10, and 13 (*n* = 10 animals). Tumor volume was shown. **b** Cleavable linker dependent anti-tumor effect of mProIFNa4-Fc. C57BL/6 J mice were s.c. inoculated with 5 × 10^5^ B16 tumor cells and i.p. treated with 1 nmol of hIg, mProIFNa4-Fc without cleavable substrate (Non-sub), or mProIFNa4-Fc on day 8 and 11 (*n* = 8 animals). Tumor volume was shown. **c**, **d** C57BL/6 J mice were s.c. inoculated with 5 × 10^5^ B16 tumor cells and i.p. treated with 1 nmol of hIg, mIFNa4-Fc, or mProIFNa4-Fc, on day 8, 11, and 14 (*n* = 10 animals). Tumor growth (**c**) and body weight (**d**) were shown. **e**, **f** C57BL/6 J mice were s.c. inoculated with 1 × 10^6^ LLC tumor cells and i.p. treated with 1 nmol of hIg, mIFNa4-Fc, or mProIFNa4-Fc, on day 12, 15, and 18 (*n* = 9 animals). Tumor growth (**e**) and body weight (**f**) were shown. **g**, **h** C57BL/6 J mice were s.c. inoculated with 1 × 10^6^ MC38 tumor cells and i.p. treated with 1 nmol of hIg, mIFNa4-Fc, or mProIFNa4-Fc, on day 9, 12, and 15 (*n* = 9 animals). Tumor growth (**g**) and body weight (**h**) were shown. Data are reported as mean ± s.e.m. Two-way ANOVA was performed to calculate *p*-values. Source data are provided as a Source Data file.

We tested the therapeutic efficacy of ProIFN in poor immunogenic and aggressive tumor models that fail to respond to various immunotherapies. In B16-bearing mice, we dosed mProIFNa4-Fc bearing a cleavable linker at 0, 0.01, 0.1, or 1 nmol. Treatment with mProIFNa4-Fc led to dose-dependent suppression in tumor growth (Fig. 3a). To test whether the proteolytic cleavage to activate mProIFNa4-Fc is required for its anti-tumor effect, we included mProIFNa4-Fc without a cleavable linker (Nsub) as a control. Non-cleavable ProIFN had a noteworthy but much weaker anti-tumor effect than cleavable ProIFN (Fig. 3b), suggesting that cleavable linker is required for the optimal anti-tumor activity of ProIFN. In both B16 and LLC models that fail to respond to various immunotherapies, mProIFNa4-Fc and unmasked mIFNa4-Fc treatment showed similar anti-tumor activities while lost weight was showed only in mIFNa4-Fc treated mice, but not in mProIFNa4-Fc treated mice (Fig. 3c–f). In the MC38 model that responds well to immunotherapies, mProIFNa4-Fc and unmasked mIFNa4-Fc also had comparable anti-tumor activities while lost weight in mice was triggered only by unmasked mIFNa4-Fc (Fig. 3g–h). These results demonstrate that mProIFNa4-Fc has substantially reduced toxicity while an uncompromised overall therapeutic effect compared to unmasked mIFNa4-Fc.

### ProIFN promotes DC cross-priming and increases tumor-specific CD8^+^ T cell response

**Fig. 4:**
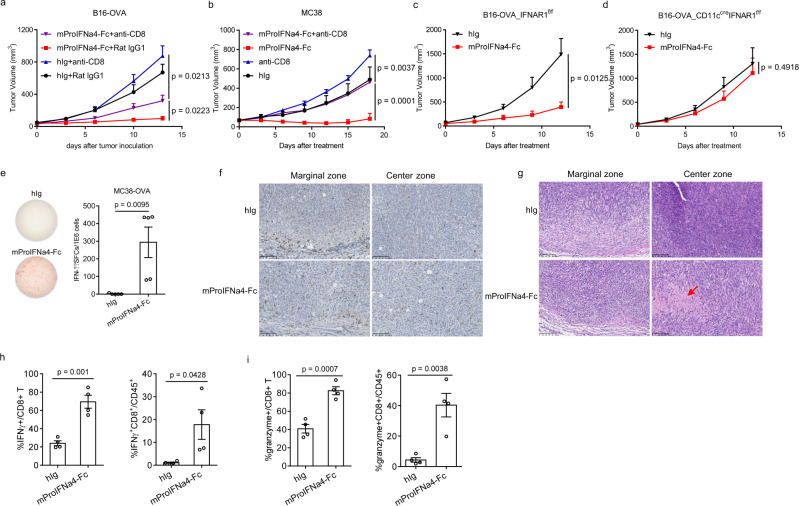
Dendritic cells and cytotoxic T cells are required for ProIFN-mediated tumor regression. **a** C57BL/6 J mice were s.c. inoculated with 5 × 10^5^ B16-OVA tumor cells and i.p. treated with 1 nmol of the fusion protein on day 8, 11, and 14 (*n* = 5 animals). 200 μg anti-CD8 was administrated on day 8, 11, and 18. Tumor growth was shown. **b** C57BL/6 J mice were s.c. inoculated with 1 × 10^6^ MC38 tumor cells and i.p. treated with 1 nmol of the fusion protein on day 9, 12, and 15 (*n* = 5 animals). 200 μg anti-CD8 was administrated on day 9, 12, and 19. Tumor growth was shown. **c** IFNAR1^f/f^ mice were s.c. inoculated with 5 × 10^5^ B16-OVA tumor cells and i.p. treated with 0.2 nmol of hIg or mProIFNa4-Fc on day 8, 11, and 14. Tumor growth was shown (*n* = 7 animals). **d** CD11c^cre^IFNAR1^f/f^ mice were s.c. inoculated with 5 × 10^5^ B16-OVA tumor cells and i.p. treated with 0.2 nmol of hIg, or mProIFNa4-Fc, on day 8, 11, and 14. Tumor growth was shown (*n* = 8 animals). **e** MC38-OVA tumor-bearing C57BL/6 mice (*n* = 5 animals) were i.p. treated with 1 nmol of hIg or mProIFNa4-Fc on day 14 and 17. Two days after the last treatment, DCs were isolated and co-cultured with isolated OT-I transgenic CD8^+^ T cells. Forty-eight hours later, IFN-γ-producing cells were enumerated by ELISPOT assay. **f**, **g** C57BL/6 J mice were s.c. inoculated with 1 × 10^6^ MC38 tumor cells and i.p. treated with 1 nmol of hIg or mProIFNa4-Fc on day 10 and 13. Tumor tissues were harvested 4 days after the last treatment. CD8^+^ IHC (**f**) and HE (**g**) staining were performed. Red arrow in the (**g**) showed the apoptosis area. The maximum unit of the scale bar is 100 µm. The images are representative of four mice samples in each group. The experiment was repeated two times independently with similar results. **h** To assess the IFN-γ producing ability of T cells, B16 tumor-bearing IFN-γ YFP reporter mice (*n* = 4 animals) were i.p. treated with 1 nmol of mProIFNa4-Fc on day 11, 14, and 17. Two days after the last treatment, tumor tissues were harvested and YFP ^+^ CD8 ^+^ T cells were determined by flow cytometry. **i** B16 tumor-bearing WT mice (*n* = 4 animals) were i.p. treated with 1 nmol of mProIFNa4-Fc on day 11, 14, and 17. Two days after the last treatment, tumor tissues were harvested and granzyme in CD8 ^+^ T cells was determined by flow cytometry. Data are reported as mean ± s.e.m. Two-way ANOVA (**a**–**d**) or two-tailed *t*-tests (**e**, **h**, **i**) were performed to calculate *p*-values. Source data are provided as a Source Data file.

IFN-I has multiple anti-tumor activities—low doses can activate innate and adaptive immunity while high doses may have other tumor suppressive activities. We hypothesized that the anti-tumor effect of ProIFN depends on DCs cross-priming and further anti-tumor T cells activating. To confirm CD8^+^ T cells are essential for the therapeutic effect of mProIFNa4-Fc, we administered a CD8-depleting antibody during treatment in B16-OVA-bearing mice or MC38-bearing mice. CD8 + T cells depletion efficacy was verified by flow cytometry (Supplementary Fig. 5). CD8^+^ cell depletion largely impaired the therapeutic effect of mProIFNa4-Fc in both B16 and MC38 tumor models (Fig. 4a, b). Mechanistically, we explored whether T cell dependency is associated with IFN targeting on DC, we selected IFNAR1^f/f^ vs CD11c^cre^IFNAR^f/f^ mice, which lack IFNAR1 on DCs (Supplementary Fig. 6). In contrast to IFNAR1^f/f^ mice that control tumor growth after ProIFN treatment, CD11c^cre^IFNAR1^f/f^ mice abolished the anti-tumor effect of mProIFNa4-Fc (Fig. 4c, d). This confirms that IFNAR signaling on DC is required for ProIFN to control tumors. To study if cross-priming function was enhanced after treatment, DCs from the draining lymph nodes (dLNs) of mProIFNa4-Fc-treated mice bearing MC38-OVA were purified and co-cultured with naive OT-I transgenic CD8^+^ T cells that express the TCR with the specificity to recognize the OVA257-264 epitope. Indeed, DCs from mProIFNa4-Fc-treated mice induced a significant increase of IFN-γ production from OT-I T cells even without additional OVA257-264 epitope in the ex vivo assay, which shows their enhanced ability to cross-prime tumor-specific CD8^+^ T cells (Fig. 4e). Immunohistochemistry data showed that CD8^+^ T cells are mainly located at the margin of tumor tissues from the control treated mice, and there were few T cells infiltrating into the tumor center area (Fig. 4f). After ProIFN treatment, there were elevated number of CD8^+^ T cells in the center zone of the tumor (Fig. 4f). Moreover, apoptotic tumor cells were found within ProIFN-treated tumor tissues (Fig. 4g), perhaps induced by effector cytokines secreted from activated CD8^+^ T cells. To assess whether ProIFN treatment increased the effector function of CD8^+^ T cells, we utilized IFN-γ YFP reporter mice to track the IFN-γ production through YFP expression. IFN-γ within tumor-CD8^+^ T cells were increased significantly after ProIFN treatment (Fig. 4h). Consistently, another effector cytokine of CD8^+^ T cells, granzyme, was also increased (Fig. 4i). These results demonstrate that ProIFN can target DC to increase the activation of CD8^+^ T cells, thus leading to tumor regression.

**Fig. 5:**
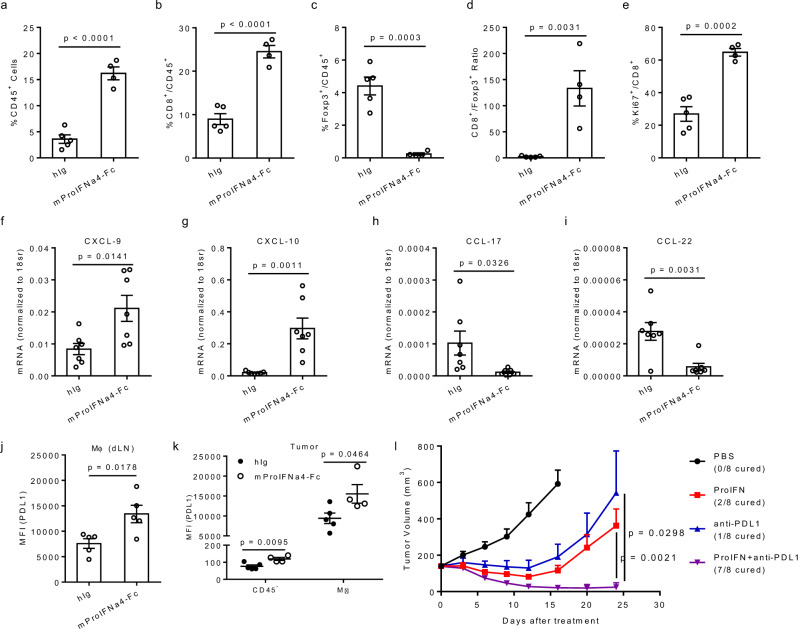
Checkpoint blockage benefits from ProIFN-optimized tumor microenvironment. **a**–**e** C57BL/6 J mice were s.c. inoculated with 5 × 10^5^ B16-OVA tumor cells and i.p. treated with 1 nmol of hIg, or mProIFNa4-Fc, on day 9, 12, and 15 (*n* = 5 animals for hIg, *n* = 4 animals for mProIFNa4-Fc). 5 days after the last treatment, mice were euthanized. Tumors and draining lymph nodes (dLNs) were extracted, digested in collagenase/DNase, and resuspended as single cells. Tumor-infiltrating T cells were analyzed via flow cytometry for the frequency of total CD45^+^ cells (**a**), CD8^+^ T cells (**b**), Foxp3^+^ T cells (**c**), CD8^+^/Foxp3^+^ ratio (**d**), and Ki67^+^CD8^+^ T cells (**e**). **f**–**i** C57BL/6 J mice were s.c. inoculated with 5 × 10^5^ B16 cells and i.p. treated with 1 nmol of hIg or mProIFNa4-Fc on day 11 (*n* = 7 animals). Tumor tissues were harvested 48 h after treatment. Intracellular RNA was extracted for RT-qPCR assay to determine expression levels of CXCL-9 (**f**), CXCL-10 (**g**), CCL-17 (**h**), and CCL-22 (**i**). **j**, **k** PD-L1 expression in tumors and draining lymph nodes (dLNs) from tumor-bearing mice in **a** was measured by flow cytometry. Mean fluorescent intensities (MFIs) of PD-L1 staining in dLN (**j**) and tumor (**k**) were shown (*n* = 5 animals). **l** Combination of mProIFNa4-Fc and a-PD-L1 antibody therapy. C57BL/6 J mice were s.c. inoculated with 1 × 10^6^ MC38 tumor cells and i.p. treated with 1 nmol of mProIFNa4-Fc on day 12, 14, and 16 (*n* = 8 animals). 200 μg anti-PD-L1 was i.p. administrated on day 12, and 16. Tumor growth was shown. Data are reported as mean ± s.e.m. Two-tailed *t*-tests (**a**–**k**) or two-way ANOVA (**l**) were performed to calculate *p*-values. Source data are provided as a Source data file.

Next, we investigated the changes of immune cell populations within tumor tissues after ProIFN treatment by flow cytometry. Gating strategy for analysis of T cells was showed (Supplementary Fig. 7). A significant increase of CD45^+^ immune cells infiltration within the tumor was found from mProIFNa4-Fc treated mice (Fig. 5a). When further probing T cell populations, we found that mProIFNa4-Fc treatment increased the percentage of CD8^+^ T cell population (Fig. 5b) but decreased the Treg population (Fig. 5c), which led to significantly increased intratumoral CD8^+^/Treg ratio (Fig. 5d). To explore whether increased expansion could contribute to CD8^+^ T cell increase within a tumor, we selected Ki67 as a representative proliferation marker. There was a clear upregulation of Ki67 expression indicating active proliferation of CD8^+^ T cells in tumors after treatment (Fig. 5e). Original dot plot data of different immune cells was also showed (Supplementary Fig. 8). IFN-I signaling can regulate T cells recruitment into tumors through chemokines. Administration of IFN-I induces a consistent production of CXCL-9 and CXCL-10 in DCs^23^. Effector T cells from adoptive T cell transfer fail to traffic to a non-T cell-inflamed melanoma model due to lack of CXCL-9/10 production^24^. On the other hand, studies show that CCL-22 leads to the recruitment of Treg into the tumor tissues^25,26^, and suppression of intratumoral CCL-22 by IFN-I inhibits migration of regulatory T cells and blocks cancer progression^27^. Therefore, we determined the expression levels of CXCL-9, CXCL-10, CCL-17, and CCL-22 in tumor tissues. ProIFN treatment significantly increased mRNA production of CD8^+^ T cell recruiting CXCL-9 and CXCL-10 but inhibited mRNA production of Treg recruiting CCL-17 and CCL-22 (Fig. 4f–i). Altogether, it shows that ProIFN can attract and increase the proliferation of CD8^+^ T cells for tumor inhibition, but decrease the immune inhibitory Tregs.

### ProIFN can improve ICB efficacy in the tumor microenvironment

PD-1/PD-L1 blockade resistance becomes a major problem for most patients^28^. We hypothesized that a lack of additional strong cross-priming may cause this relapse. As ProIFN treatment can increase cross-priming to reactivate T cells, we proposed that it can improve immune checkpoint blockade (ICB) efficacy. Meanwhile, IFN can upregulate PD-L1 on treated cells. PD-L1 can be increasingly expressed on antigen-presenting cells (APCs) or tumor cells. To determine which cell subsets have higher PD-L1 expression after treatment, we collected tissues from B16-OVA tumor-bearing mice after mProIFNa4-Fc treatment and the PD-L1 expression profiles were accordingly evaluated by flow cytometry. The gating strategy for the analysis of macrophages and dendritic cells was showed (Supplementary Fig. 9). Interestingly, mProIFNa4-Fc treatment upregulated PD-L1 expression on myeloid cells within dLN (Fig. 5j). In the tumor microenvironment, myeloid cells expressed much higher level of PD-L1 than tumor and stromal (CD45^-^) cells, and mProIFNa4-Fc treatment increased PD-L1 expression on both CD45^–^ cells and myeloid cells (Fig. 5k). The data implied a potential resistance mechanism to ProIFN due to an increase in this negative PD-L1 signal. We then tested whether mProIFNa4-Fc can synergize with anti-PD-L1 in advanced tumors. While mProIFNa4-Fc and anti-PD-L1 by themselves can only delay tumor progression, treatment with both showed a strong synergistic anti-tumor effect and an increased percentage of tumor-free mice (Fig. 5l). These results demonstrate that ProIFN improves ICB efficacy in advanced tumors.

### ProIFN can improve radiation efficacy in both primary and metastatic tumors

**Fig. 6:**
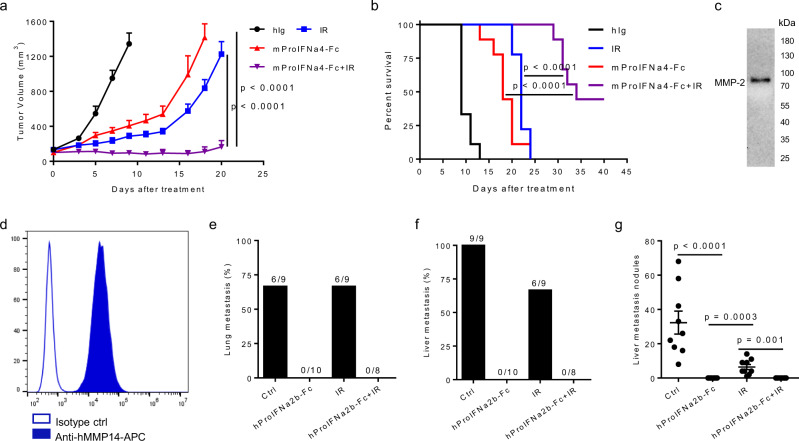
ProIFN improves radiation efficacy. **a**, **b** C57BL/6 J mice were s.c. inoculated with 5 × 10^5^ B16 tumor cells and i.p. treated with 1 nmol (8 mg/kg) of mProIFNa4-Fc on day 10, 13, and 16 (*n* = 9). Tumors were locally received a single 30-Gy dose on day 9. Tumor growth (**a**) and survival curve (**b**) were shown. **c** Western blot analysis of MMP-2 expression level from the culture supernatant of MDA-MB-231 cells. The experiment was repeated twice independently with similar results. **d** Flow cytometry analysis of MMP-14 expression level on MDA-MB-231 cells. **e**–**g** Human CD34^ +^ hematopoietic stem cells transferred NSG-SGM3 mice were s.c. inoculated with 2 × 10^6^ MDA-MB-231 tumor cells and i.p. treated with 10 mg/kg hProIFNa2b-Fc on day 18, 21, 24, 31, 40, and 47 (*n* = 9 animals for Ctrl, *n* = 10 animals for hProIFNa2b-Fc, *n* = 9 animals for IR, *n* = 8 animals for hProIFNa2b-Fc + IR). Tumors were locally received a single 12-Gy dose on day 17. Mice were euthanized on day 57. Lung metastasis percentage (**e**), liver metastasis percentage (**f**), and number of liver metastasis nodules were shown (**g**). Data are reported as mean ± s.e.m. Two-way ANOVA (**a**), the logrank test, (**b**) or two-tailed *t*-tests (**g**) were performed to calculate *p*-values. Source data are provided as a Source Data file.

Radiation therapy (RT) is widely used in killing tumor cells and release tumor antigens, but relapse becomes a major clinical problem. We proposed that additional exogenous IFN-I can increase the capturing and processing of tumor antigen for better T cell expansion that prevents relapse after RT. To test this hypothesis, we evaluated whether ProIFN can enhance tumor regression. In B16-bearing mice, tumors started to relapse from two weeks after treatment in either mProIFNa4-Fc- or IR-treated group. However, mice treated with both effectively controlled tumor relapse (Fig. 6a) and substantially increased survival (Fig. 6b). Therefore, ProIFN can improve RT efficacy.

Metastasis is another common issue in the late stages of cancer. The potent anti-tumor effect of ProIFN led us to explore whether ProIFN could assist IR in controlling metastatic tumors. We used a humanized mice model, in which human CD34^+^ hematopoietic stem cells were transferred into NSG-SGM3 mice. These humanized mice partially restored the number and function of human immune cells. MDA-MB-231 cells are able to colonize in the bone, liver, lung, adrenal glands, ovary, and brain after intravenous injection^29^. MMP-2 secretion by MDA-MB-231 cells was verified by western blot analysis of cell culture supernatant (Fig. 6c). MMP-14 expression on MDA-MB-231 cells was verified by flow cytometry (Fig. 6d). Indeed, the IR treatment showed moderate suppression without any metastasis-free mice. Impressively, hProIFNa2b-Fc treatment successfully prevented both lungs and livers from tumor metastasis, and it was striking 100% protection against metastasis. Mice treated with the combination of IR and hProIFNa2b-Fc achieved the same results with hProIFNa2b-Fc treatment (Fig. 6e–g). These data demonstrate that the administration of ProIFN can improve IR efficacy in both primary and metastatic tumors.

### ProIFN pharmacokinetics and tolerability in non-human primates

To evaluate whether we could translate our mouse studies to human studies, we tested several preclinical studies of hProIFNa2b-Fc. The high stability of ProIFN is an important property for wider and more reliable translational use. We tested whether hProIFNa2b-Fc could maintain stability and activity in multiple conditions. Stability studies showed that hProIFNa2b-Fc was able to be kept at 5 °C for at least 6 months, 25 °C for 6 months, and 40 °C for 6 weeks without apparent loss of activity determined by IFN-I activity reporter assay (Supplementary Table 2). Short half-life is one of the major shortcomings for free recombinant IFN. The pharmacokinetics of hProIFNa2b-Fc were determined in rhesus monkeys. Following a single s.c. administration, serum hProIFNa2b-Fc levels increased slowly over time, reaching maximum serum levels 16–20 h after dosing in all groups of monkeys. hProIFNa2b-Fc exhibited a very long terminal half-life, ranging from 65 to 103 h. Dose-related increase in C_max_ was observed following injection of hProIFNa2b-Fc (Supplementary Table 3).

We investigated the potential safety benefits conferred by this tumor-targeting strategy. In acute toxicity study, cynomolgus monkeys were s.c. administrated with single dose of hProIFNa2b-Fc at 20, 40, or 60 mg/kg, respectively. The MTD (Maximum Tolerated Dose), based on clinical observation, was 60 mg/kg (830.45 nmol IFN/kg). None of the monkeys in ProIFN-treated groups died over the duration of the acute toxicity study including the 60 mg/kg treated group (Supplementary Table 4). In a repeat-dose toxicity study, rhesus monkeys were s.c. administrated every 4 days for 29 consecutive days with hProIFNa2b-Fc at doses of 0.2, 0.6, or 2.0 mg/kg, respectively. The NOAEL (No-Observed-Adverse-Effect Level), based on preclinical observation, was 2 mg/kg (27.68 nmol IFN/kg). All ProIFN-treated monkeys didn’t have any death or decline in health to the point of requiring sacrifice over the duration of the repeat-dose toxicity study (Supplementary Table 5).

## Discussion

The systemic administration of IFN-I is accompanied by major adverse outcomes, which have prompted attempts to create tumor-targeting deliveries of IFN-I. Recently, antibody prodrugs have been studied by different masking strategies to improve the safety profile of therapeutic antibodies^30–32^. Here, we have described a natural approach for conditional IFN-I masking, in which the extracellular domain of IFNAR reversibly blocks IFN activity. This study demonstrates the following advantages and features of ProIFN in cancer therapy. (1) Host-self natural receptor masks are readily useable without requiring extensive screening to identify a foreign peptide mask. (2) ProIFN can avoid the toxicities associated with systemic administration of free IFN. (3) Safety-permitted high dose administration of ProIFN ensures its potency. (4) Therapeutic effects of ProIFN depend on innate and adaptive immunity. (5) ProIFN can increase CD8^+^ T cells in the tumor microenvironment, and thus can potentially be used in cold tumors. (6) ProIFN can improve ICB efficacy and IR efficacy. (7) This prodrug can even eliminate spontaneous metastases.

IFNAR2 is the primary binding protein and binds IFN-I molecules with high affinity; IFNAR1 subsequently binds the molecule with low affinity^33^. Our results demonstrate that the extracellular domain of human IFNAR2 is a better mask than IFNAR1, which correlates with their binding affinity. Activation of ProIFN by the cleavage of the substrate linker depends on tumor-enriched enzymes. In this case, our selection of proteases was based on their expression levels in tumors and normal tissues. MMP-14 is impressive for its widely high expression in all tested tumors. As MMP-14 is the physiologic activator of latent MMP-2, a higher amount of MMP-14 usually results in increased MMP-2 activity^34^. Therefore, substrates that are sensitive to both MMP-14 and MMP-2 were selected from literature reports. The tumor targeting of ProIFN allows us to administer higher doses to achieve a therapeutic window without major adverse outcomes.

While the therapeutic effect of local delivery of IFN-I is dependent on adaptive immunity by increasing antigen cross-presentation^16^, it is unclear if tested systemically delivered doses of ProIFN can have strong effects on cross-presentation and T cell expansion. Our data also showed that ProIFN targeted DC for increasing cross-priming of T cells, thus leading to tumor regression. Impressively, we observed an increase in CD8^+^ T cells but a decrease in Tregs in tumors, which resulted in a dramatic high CD8^+^/Treg ratio. This could potentially be that IFN-I signaling affects the T cells recruitment into tumors. The mechanism here we showed was that ProIFN upregulated CXCL-9 and CXCL-10, while downregulated CCL-17 and CCL-22. These changes of produced chemokines could result in more CD8^+^ T cells and less Tregs trafficking to the tumor microenvironment. These data are consistent with previous studies^24–27^. Although immune checkpoint blockade antibodies have changed the treatment landscape of many tumors, the response rate remains relatively low in most cases. Cold tumors which lack intratumoral immune cell infiltration is a major factor to blame^35^. It is likely that ProIFN can increase CXCL-9/10 inside TME for better T cell recruitment. Our data proved that directing exogenous IFN-I to the tumor microenvironment by ProIFN turned cold tumors into hot tumors by increasing infiltration of CD45^+^ cells including more CD8^+^ T cells but fewer Tregs. These studies may explain the dual function of ProIFN treatment on the modified numbers of different T cells leading to increase CD8^+^/Treg ratio. In addition, APCs highly expressed PD-L1, and ProIFN treatment further upregulates PD-L1 expression on macrophages. These results prompted us to combine ProIFN with anti-PD-L1, which controlled tumor growth synergistically. Therefore, checkpoint blockade together with ProIFN have the potential to improve the clinical response rate in patients with cold tumors.

More than 50% of patients with advanced cancer often receive IR to reduce their tumor burden^36^. While IR can rapidly release antigens, remaining tumors might relapse. We proposed to use exogenous IFN-I to capture those antigens to control remaining tumors. Our data showed that combination treatment of IR with ProIFN synergistically controlled tumor growth, increase anti-tumor immunity, and reduced tumor relapse. Metastasis is a major cause of mortality for various cancer patients. In a mouse cancer model, administration of IFN-I led to reduced bone metastases and prolonged survival, while metastasis was accelerated in IFNAR deficient mice^37^. Consistent with this study, our data showed that human ProIFN strongly suppressed a triple-negative breast cancer line from metastasizing to lungs and livers in a humanized mice model, either as a monotherapy or combination therapy with IR. In fact, clinical trials have shown that some patients with high-risk melanoma, a highly refractory solid malignancy, that receive IFN-I treatment following surgery demonstrated an improved relapse-free survival rate^38^. We propose that ProIFN can be a powerful drug to improve IR efficacy in tumor recurrence and metastatic outgrowth in advanced cancers. Importantly, the advantage of low toxicity with ProIFN in vivo may allow clinicians to use higher dose and longer-term treatment, which could achieve a better anti-tumor effect than with current unmasked forms.

Peg-IFN is created by covalently bonding the IFN molecule with polyethylene glycol, resulting in a compound with sustained absorption and a longer half-life than free recombinant IFN. The pharmacokinetics study of human ProIFN in monkeys shows that ProIFN reaches maximum serum levels 16–20 h after administration, which is much longer compared to 4.5 – 5.5 h of Peg-IFN. The half-life of ProIFN is ranging from 65 to 103 h, which is also much longer compared to 29.2–34.1 h of Peg-IFN^39^. Free IFN has a very short terminal half-life of 3.5 h^40^. Peg-IFN has increased half-life compared to free IFN. Our data shows that ProIFN has further prolonged circulation half-life than Peg-IFN. In addition, Peg-IFN cannot reduce the incidence of AEs in patients compared to free IFN, due to its lack of tumor-targeting capabilities. The improvement in safety for ProIFN is due to its tumor targeting, despite more drug exposure in vivo. The MTD of hProIFNa2b-Fc was 830.45 nmol IFN/kg, which is ~5.2 times that of Peg-IFN (158.23 nmol IFN/kg) in monkeys. The monkey could tolerate well to 830.45 nmol IFN/kg of ProIFN, while both female monkeys from the high-dose (316.5 nmol IFN/kg) Peg-IFN treated group had early deaths on the study. In repeat-dose toxicity study, the NOAEL was 27.68 nmol IFN/kg, which is ~7.3 times that of Peg-IFN (3.81 nmol IFN/kg) in monkeys. These results show the much safer and more tolerable nature of human ProIFN as the next-generation IFN variant.

Overall, we report an IFN-I variant that is masked as a prodrug by its natural receptor. Our study demonstrates an effective tumor-activating IFN-I, which increases immunity against tumors with minimal toxicity in the host. Most importantly, this prodrug can improve checkpoint blockade therapy efficacy, IR efficacy, and control metastasis, which are clinically closely relevant and difficult to treat in advanced cancers. This strategy and design of ProIFN can also apply to other cytokines or proteins which have been limited by the unwanted toxicities of systemic stimulation. Taken together, we provide both an immune-activating therapy for tumors and an effective strategy for designing unique and safe cancer treatments.

## Methods

### Mice

Female C57BL/6 J mice, B6.Cg-Tg(Itgax-cre)1-1Reiz/J (Cd11c-Cre) mice and NOD.Cg-Prkdc^scid^ Il2rg^tm1Wjl^ Tg(CMV-IL3, CSF2, KITLG)1Eav/MloySzJ (NSG-SMG3) mice were purchased from The Jackson Laboratory. Ifnar1flox/flox mice were kindly provided by Dr. Ulrich Kalinke of the Institute for Experimental Infection Research. All mice were maintained under specific pathogen-free conditions. Animal care and experiments were carried out under institutional and National Institutes of Health protocol and guidelines. This study has been approved by the Institutional Animal Care and Use Committee of the University of Texas Southwestern Medical Center.

### Cell Lines and Reagents

B16, LLC, MC38, and MDA-MB-231 cells were purchased from ATCC. 293-Dual™ hSTING-R232 cells were purchased from InvivoGen. RAW-Lucia ISG cells were obtained from Dr. Zhijian J. Chen of University of Texas Southwestern Medical Center. B16-0VA and MC38-OVA cells were made by lentiviral transduction of the OVA gene. All cell lines were routinely tested using a mycoplasma contamination kit (R&D) and cultured in Dulbecco’s modified Eagle’s medium supplemented with 10% heat-inactivated fetal bovine serum, 100 U/ml penicillin, and 100 U/ml streptomycin under 5% CO_2_ at 37 °C. Anti-CD8 (53–5.8) was purchased from BioXCell. Goat anti-human IgG-HRP (sc-2453) was purchased from Santa Cruz. Anti-PD-L1 (Atezolizumab) was kindly provided by UT Southwestern Simmons Cancer Center Pharmacy.

### Production of fusion proteins

For IFN-Fc construction, human IFNa2 or murine IFNa4 cDNA was fused to the N-terminal of human IgG1 Fc via a GGG linker in the pEE6.4 vector (Lonza). For ProIFN construction, extracellular domains of human or murine IFNAR1/2 were fused to the N-terminal of IFN-Fc via a GGGGS-SGQLLGFLTA-GGGGS (substrate) linker or a GGGGSGGGGS (non-substrate) linker in the pEE6.4 vector, respectively. Fusion proteins were expressed via transient transfection of FreeStyle 293-F cells and were purified using CaptivA^®^ Protein A Affinity Resin according to the manufacturer’s manual (Repligen). The heterogeneity and purity were confirmed by SEC-HPLC and CE-SDS (Supplementary Table 1).

### Detection of endotoxin in fusion proteins

Endotoxin was measured via ToxinSensorTM Chromogenic LAL Endotoxin Assay Kit (GenScript) according to the manufacturer’s instructions. For all fusion proteins, the amount of endotoxin was determined to be <10 endotoxin units (EU)/mg fusion protein.

### In vitro proteolysis of ProIFN

Human MMP-14 was activated via incubation with 0.1 µg/mL rhTrypsin 3 at 37 °C for 1 h. Stop reaction with 1 mM AEBSF and incubate for 15 min at room temperature. Human or mouse MMP-2 was activated via incubation with 1 mM APMA at 37 °C for 1 h. For in vitro cleavage, ProIFN (20 μg at 200 ug/mL) was incubated at 37 °C overnight (~16 h) with activated human MMP-14 at 2 ug/mL in assay buffer (50 mM Tris, 3 mM CaCl_2_, 1 µM ZnCl_2_, pH 8.5), or activated MMP-2 at 1 ug/mL in assay buffer (50 mM Tris, 10 mM CaCl_2_, 150 mM NaCl, 0.05% (w/v) Brij 35, pH 7.5).

### IFN-I activity reporter assay

Human IFN-I activity was measured in 293-Dual™ hSTING-R232 cells. Murine IFN-I activity was measured in RAW-Lucia ISG cells. Sample dilutions were prepared and added by 50 μl per well of a flat-bottom 96-well plate. Prepare a cell suspension of 293-Dual™ hSTING-R232 cells at 1 × 10^6^ cells per ml in cell culture medium, or prepare a cell suspension of RAW-Lucia ISG cells at 2 × 10^6^ cells per ml in cell culture medium. Add 50 μl of cell suspension per well and incubate the plate at 37 °C in a CO_2_ incubator for 20–24 h. The supernatant of cell culture medium was used to test human IFN-I activity by QUANTI-Blue™ solution (InvivoGen) via secreted embryonic alkaline phosphatase activity, or murine IFN-I activity by QUANTI-Luc™ solution (InvivoGen) via secreted coelenterazine-utilizing luciferase activity.

### Enzyme-linked immunosorbent assay (ELISA)

Microtiter plates (Corning Costar) were coated with capture antibody (goat anti-human IgG, Fcγ fragment specific) overnight at 4 °C. After washing and blocking, diluted plasma samples were added and incubated at room temperature for 1 h. After washing, HRP-conjugated goat anti-human IgG (H + L) was added and incubated at room temperature for 1 h. After washing, add TMB substrate solution. Measure absorbance after adding stop solution using the SPECTROstar Nano (BMG LABTECH).

### Pharmacokinetics of mouse ProIFN

The purified mProIFNa4-Fc or mIFNa4-Fc were i.v. injected a dose of 1 nmol in female C57BL/6 J mice (four animals per group, randomly assigned). The blood was drawn into K_2_EDTA tubes via the submandibular vein (cheek punch) at various time points and processed to plasma. The fusion protein concentration at each time point was calculated by ELISA.

### Tumor growth and treatments

A total of 5 × 10^5^ B16, 5 × 10^5^ B16-OVA, 1 × 10^6^ LLC, 1 × 10^6^ MC38, 1 × 10^6^ MC38-OVA, or 2 × 10^6^ MDA-MB-231 tumor cells were s.c. injected into the flank of mice. Tumor volumes were measured by length (a) and width (b) and calculated as tumor volume = ab^2^/2. Tumors, allowed to grow for 7–14 days to reach 50–100 mm^3^, were i.p. treated with fusion proteins. For CD8 depletion, 200 μg of anti-CD8 antibody was i.p. injected for three doses. For PD-L1 blockade combination therapy, 200 μg of anti-PD-L1 antibody was i.p. injected for two doses.

### Humanized mouse model

Humanized mouse reconstitution was previously described^41^. Briefly, human CD34 + cells purified from cord blood (UT Southwestern Parkland Hospital) were intravenously injected into female NSG-SGM3 mice which were irradiated with 100 cGy (X-ray irradiation with X-RAD 320 irradiator) 1 day prior to transfer. Mice were maintained for about 12 weeks to allow human immune reconstitution. Experiments were performed in compliance with UTSW Human Investigation Committee protocol and UTSW Institutional Animal Care and Use Committee.

### RNA extraction and quantitative real-time PCR

Total RNA of collected tissues were extracted with TRIzol RNA Isolation Reagents (Thermo-Invitrogen) and reverse-transcribed with Script™ gDNA Clear cDNA Synthesis Kit (Bio-Rad). Real-time PCR was performed with SsoAdvanced Universal SYBR Green Supermix (Bio-Rad) according to the manufacturer’s instructions and with different primer sets on CFX Connect Real-Time PCR Detection System (Bio-Rad). The primers used for Real-time PCR were shown in Supplementary Table 6. The 2^–ΔΔCt^ method was used to calculate relative expression changes.

### Flow cytometry analysis

Tumor tissues or dLNs were collected and digested with 1 mg/mL Collagenase I (Sigma) and 0.5 mg/mL DNase I (Roche) at 37 °C for 30 mins. Single-cell suspensions were obtained via passing through a 70 μm cell strainer. Cells were incubated with anti-FcγIII/II receptor (clone 2.4G2) for 15 mins to block non-specific binding, and then incubated with indicated antibodies (supplementary table 6) for 30 min at 4 °C in the dark. 7-AAD Viability Staining Solution was used to exclude dead cells. Foxp3 and Ki67 were stained intracellularly by using True-Nuclear transcription factor buffer set (BioLegend) following the manufacturer’s instructions. BD™ CBA Mouse Inflammation Kit (BD Biosciences) was used to quantitatively measure cytokines in mice plasma samples according to the manufacturer’s protocol. Data were collected on CytoFLEX flow cytometer (Beckman Coulter, Inc) and analyzed by using CytExpert (Beckman Coulter, Inc) or FlowJo (Tree Star Inc., Ashland, OR) software.

### Ex vivo dendritic cell cross-presentation

MC38-OVA tumor-bearing mice were i.p. treated with hIg or mProIFNa4-Fc on day 14 and 17. Two days after the last treatment, DCs were isolated from dLNs via by EasySep Mouse CD11c Positive Selection Kit (STEMCELL Technologies). Approximately 2.5 × 10^4^ DCs were co-cultured with 2 × 10^5^ purified OT-I transgenic CD8^+^ T cells to restimulate the T cells. After 48 h culture, ELISPOT assay was performed using the IFN-γ ELISPOT kit (BD Bioscience) according to the manufacturer’s instructions. IFN-γ spots were enumerated via CTL-ImmunoSpot^®^ S6 Analyzer (Cellular Technology Limited).

### Histology and tissue analysis

Formalin-fixed tissues were embedded in paraffin and cut into 5 μm sections. Sections were evaluated by H&E and immunohistochemical analysis using antibodies specific for CD8 (Cell Signaling, 98941). Following an initial antigen retrieval with Tris-EDTA-glycerol (10%) buffer (Thermo Fisher Scientific) and inhibition of endogenous peroxidase activity, the slides were incubated with primary antibody overnight at 4 °C. Slides were then incubated with alkaline phosphatase-conjugated secondary antibody (ImmPRESS-AP anti-rabbit IgG alkaline phosphatase [catalog MP-5401]) for 1 h at 25 °C. The following coloring was developed using the DAB chromogenic substrate. Color images were obtained with Hamamatsu Nanozoomer 2.0HT (UTSW Whole Brain Microscopy Facility). Pictures were analyzed using NDP.view2 software.

### Preclinical studies of human ProIFN in monkeys

All animals were housed, maintained, and treated in accordance to standard ethical animal handling guidelines. The study was reviewed and approved by the IACUC at WuXi AppTec. For pharmacokinetics study, rhesus monkeys (*n* = 6, 3/sex/group) were s.c. administrated with single dose of hProIFNa2b-Fc at 0.5, 1.5, and 5 mg/kg, respectively. Blood samples were collected at 0 (pre-dose), 3, 6, 12, 24, 36, 48, 60, 72, 96, 120, 168, 216, 264, and 336 h post-dose. Concentrations of hProIFNa2b-Fc in serum samples were determined by ELISA. For acute toxicity study, cynomolgus monkeys (*n* = 2, 1/sex/group) were s.c. administrated with single dose of hProIFNa2b-Fc at 20, 40, or 60 mg/kg, respectively. Observations for overt signs of clinical toxicity were performed within 4 h after dosing. General clinical observations were performed every morning and afternoon for 14 consecutive days. For repeat-dose toxicity study, rhesus monkeys (*n* = 10, 5/sex/group) were s.c. administrated every 4 days with hProIFNa2b-Fc at doses of 0.2, 0.6 or 2.0 mg/kg, respectively. Treatments continued for 29 days before the 28 days treatment-free recovery period. Criteria for evaluation included viability (morbidity/mortality), clinical observations, injection site observation, body weight, food consumption, body temperature, ophthalmic examinations, clinical pathology (hematology, serum chemistry, coagulation, urinalyses), safety pharmacology (blood pressure, heart rate, electrocardiogram, respiratory and nervous system), immunological analysis (B/T lymphocyte typing, cytokine, and immunoglobulin analysis), anti-drug antibody analysis, neutralizing antibody analysis, organ weight, and histopathological evaluation.

### Statistical analysis

All the data analyses were performed with GraphPad Prism statistical software and shown as mean ± s.e.m. *P*-value was determined by two-way ANOVA for tumor growth, the logrank test for survival analysis, or unpaired two-tailed *t*-tests for other analysis. P-values considered significant as follows: *P < 0.05; **P < 0.01; ***P < 0.001 and ****P < 0.0001.

### Reporting summary

Further information on research design is available in the Nature Research Reporting Summary linked to this article.

## Supplementary information

Supplementary information

Reporting Summary

Peer Review Fil

## Data Availability

The Human MMP expression level publicly available data used in this study are available in the TIMER (Tumor IMmune Estimation Resource) under the Diff Exp module [https://cistrome.shinyapps.io/timer/]. Source data are available as a Source Data file. The remaining data are available within the Article or Supplementary information. Source data are provided with this paper.

## Source data

Source Data
