# Supplementary material for: Next generation of tumor-activating type I IFN enhances anti-tumor immune responses to overcome therapy resistance
Source: Nat Commun. 2021 Oct 7;12:5866. doi: 10.1038/s41467-021-26112-2 (PMC8497482; doi:10.1038/s41467-021-26112-2)
Supplement: Supplementary file 1 — Supplementary information [file 41467_2021_26112_MOESM1_ESM.pdf]

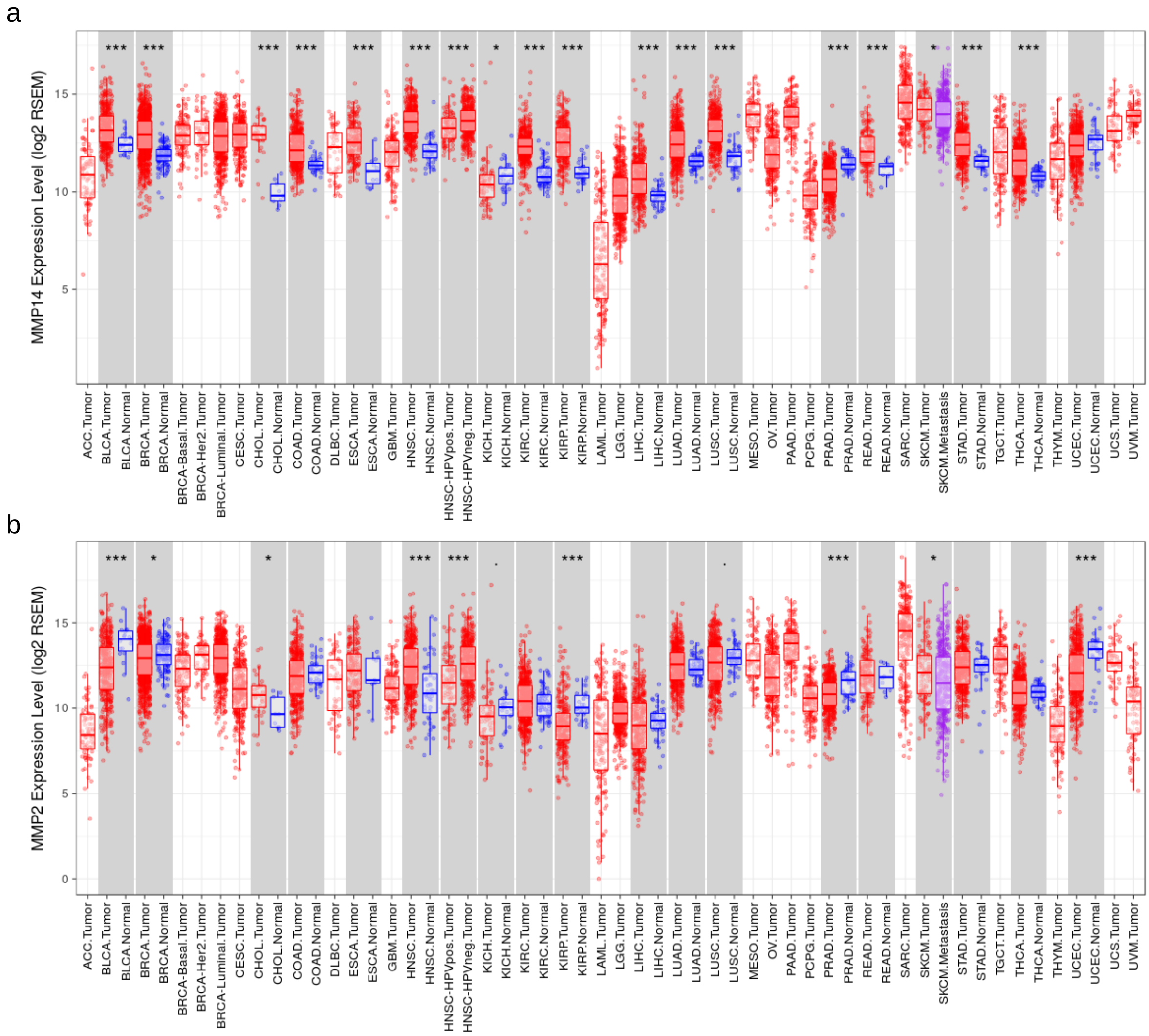

Supplementary Figure 1. Human MMP expression level between tumor and adjacent normal tissues. The DiffExp module of TIMER (Tumor IMmune Estimation Resource) website online analysis of the comparison of MMP-14 (a) and MMP-2 (b) expression levels for all samples from TCGA (The Cancer Genome Atlas). Red dots indicate tumor tissues, and blue dots indicate adjacent normal tissues.

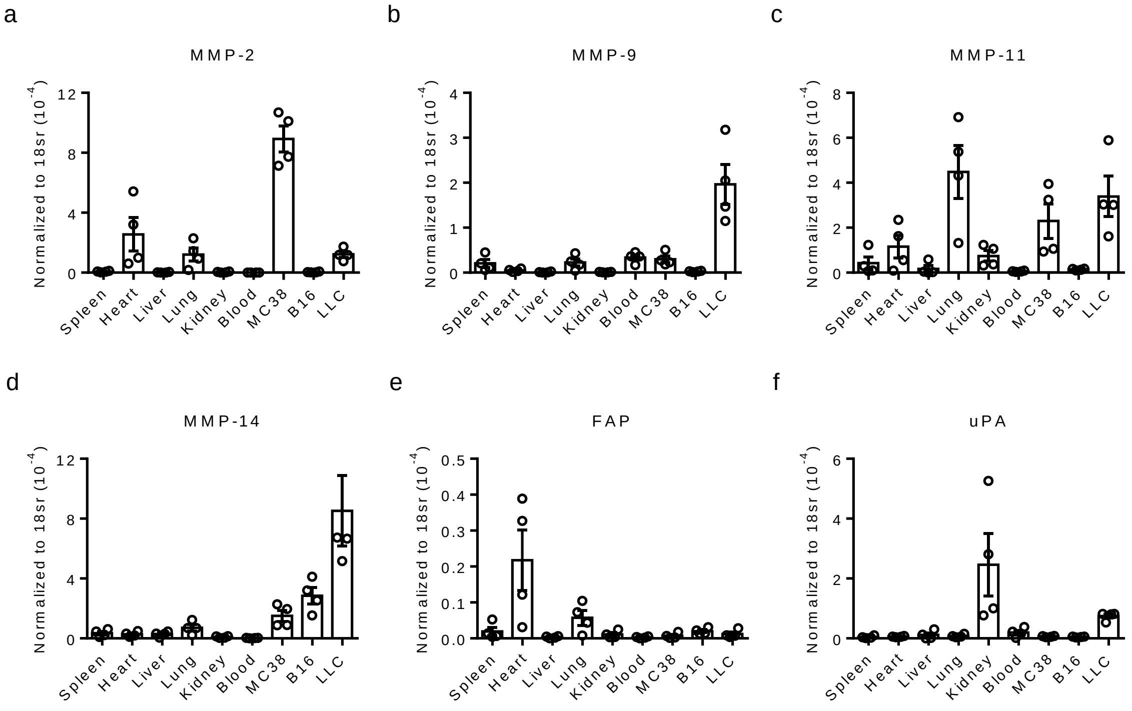

Supplementary Figure 2. MMPs expression levels in mouse normal tissues and tumors. C57BL/6J mice were s.c. inoculated with 1×10^6 MC38 cells, 5×10^5 B16 cells, or 1×10^6 LLC cells (n=4 animals). Indicated normal tissues and tumors were harvested between day 11 and day 18. Intracellular RNA was extracted for RT-qPCR assay to determine expression levels of MMP-2 (a), MMP-9 (b), MMP-11 (c), MMP-14 (d), FAP (e), and uPA (f). Data are reported as mean ± s.e.m. Source data are provided as a Source Data file.

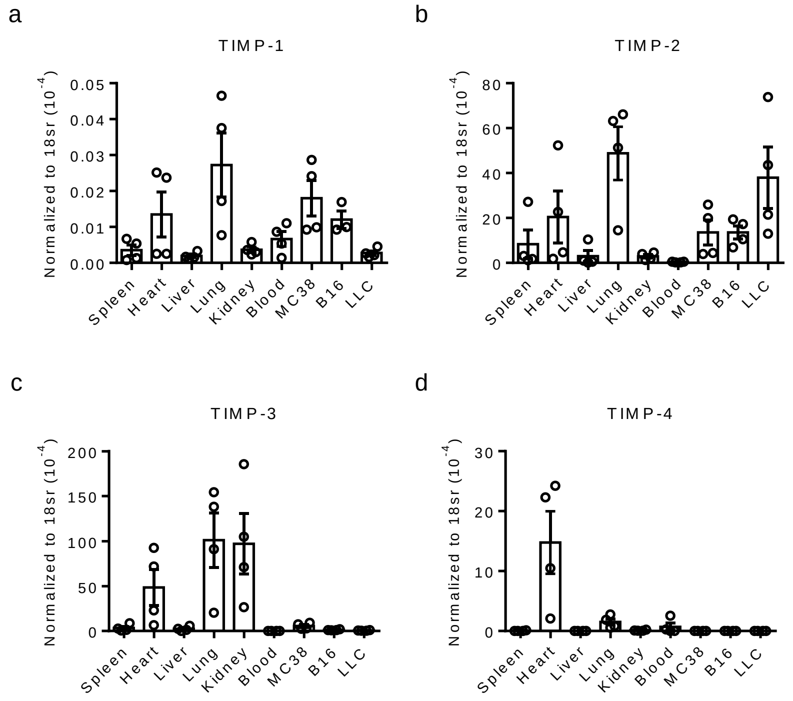

Supplementary Figure 3. TIMPs expression levels in mouse normal tissues and tumors. C57BL/6J mice were s.c. inoculated with 1×10^6 MC38 cells, 5×10^5 B16 cells, or 1×10^6 LLC cells (n=4 animals). Indicated normal tissues and tumors were harvested between day 11 and day 18. Intracellular RNA was extracted for RT-qPCR assay to determine expression levels of TIMP-1 (a), TIMP-2 (b), TIMP-3 (c), and TIMP-4 (d). Data are reported as mean ± s.e.m. Source data are provided as a Source Data file.

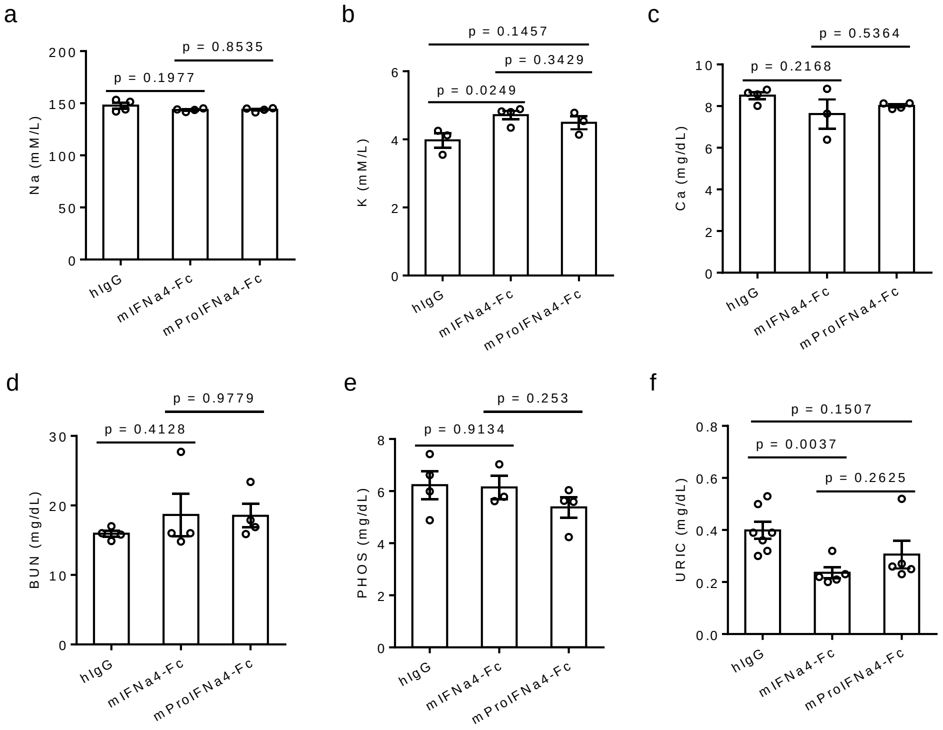

Supplementary Figure 4. Effects of mIFNa4-Fc or mProIFNa4-Fc treatments on kidney functional parameters. (a-f) Healthy C57BL/6J mice were intraperitoneally (i.p.) treated with 1 nmol of hIg, mIFNa4-Fc, or mProIFNa4-Fc, every 3 days for 3 times (n=4 animals). Plasma samples were collected 2 days after the last treatment. The levels of sodium Na (a), potassium K (b), calcium Ca (c), urea BUN (d), inorganic phosphorus PHOS (e), and uric acid URIC (f) were determined by UTSW Metabolic Phenotyping Core. Data are reported as mean ± s.e.m. Two-tailed t-tests were performed to calculate *p* values. Source data are provided as a Source Data file.

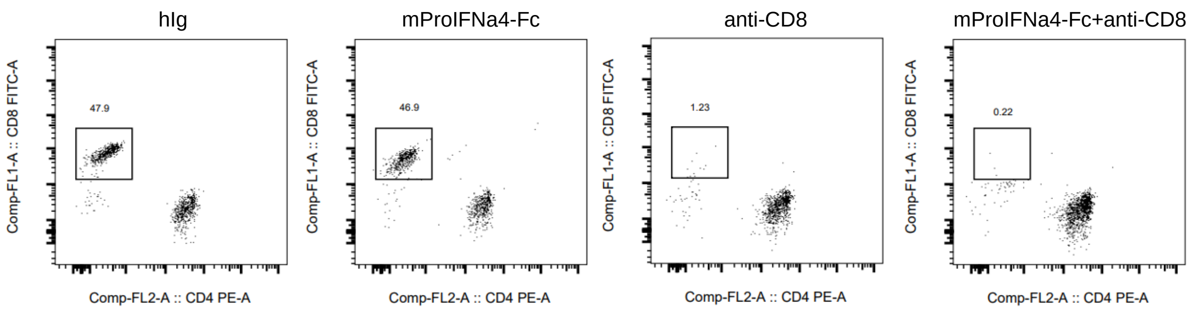

Supplementary Figure 5. CD8$^+$ T cells depletion by anti-CD8 antibody. Tumor-bearing mice were i.p. treated with 1 nmol of fusion protein on day 8, 11, and 14 (n=5). 200 µg anti-CD8 was administrated on day 8, 11, and 18. The depletion of CD8$^+$ T cells in the peripheral blood were verified by FACS at day6 after first treatment.

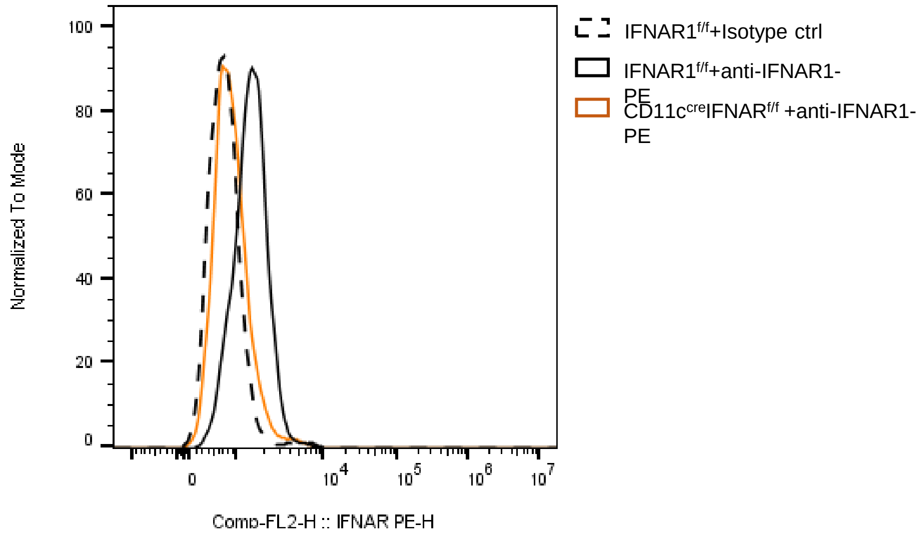

Supplementary Figure 6. IFNAR1 expression level on DC cells of CD11c^{cre}IFNAR$^{f/f}$ mice. IFNAR$^{f/f}$ or CD11c^{cre}IFNAR$^{f/f}$ mice were euthanized. Spleens were extracted, digested in collagenase/DNAse, and resuspended as single cells. The IFNAR1 expression level on DC cells were analyzed via flow cytometry.

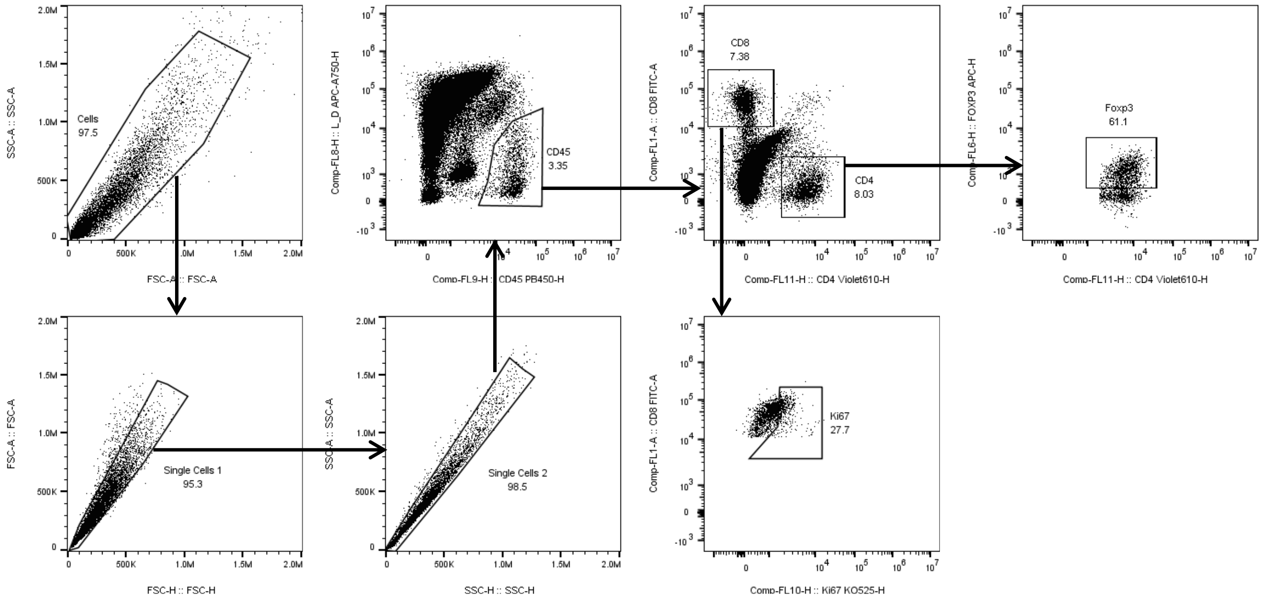

Supplementary Figure 7. Gating strategy for analysis of T cells from B16-OVA tumor tissues presented on Fig.5a-5e.

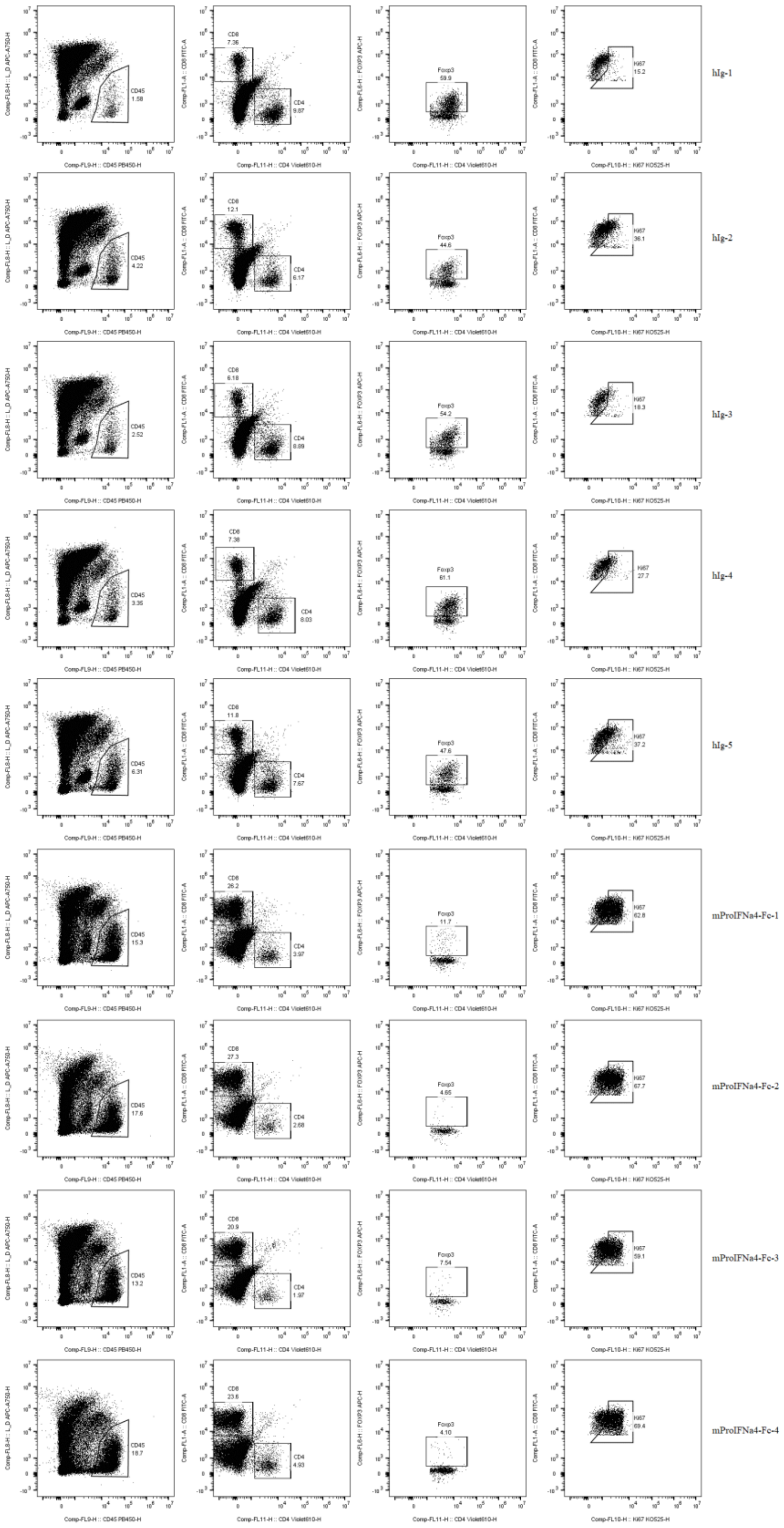

Supplementary Figure 8. Original dot plot data of different immune cells from B16-OVA tumor tissues presented on Fig.5a-5e.

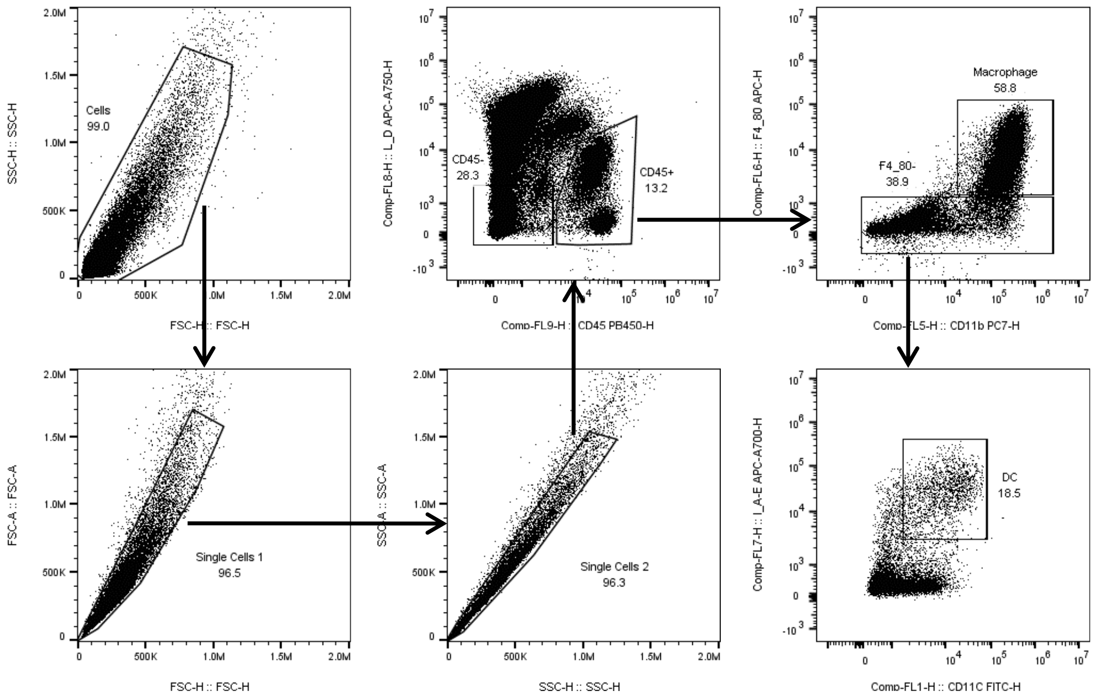

Supplementary Figure 9. Gating strategy for analysis of macrophages and dendritic cells from B16-OVA tumor tissues presented on Fig.5j-5k. Macrophage was identified as $CD45^+CD11b^+F4/80^+$, DC was identified as $CD45^+CD11c^+MHCII^+$.

Supplementary Table 1. Quality specification of hProIFNa2b-Fc

Category	Test	Result
Purity	SEC-HPLC	Monomers: 98.6%
		Aggregates: 0.7%
		Fragments: 0.6%
	Non-reduced CE-SDS	Monomers: 100%
		Fragments: ND
	Reduced CE-SDS	99.3%
Safety	Endotoxin	<10 EU/mg

ND, not detectable.

Supplementary Table 2. Stability study of hProIFNa2b-Fc

Study	Storage	Duration	Completed	Conclusion
Long-term	5 ± 3 ℃	0, 3, 6 Months	6 Months	Conforms to specification, no significant change compared with release testing.
Accelerated	25 ± 2 ℃	0, 1, 2, 3, 6 Months	6 Months	Conforms to specification, no significant change compared with release testing.
Stress Testing	40 ± 2 ℃	0, 1, 2, 4, 6 Weeks	6 Weeks	Residual Moisture showed slight uptrend compared with release testing, but still conforms to the specification.

Supplementary Table 3. Pharmacokinetics of hProIFNa2b-Fc in rhesus monkeys

Parameter	Unit	hProIFNa2b-Fc (n=6)			Peg-IFN (n=4)		
Dose (s.c.)	nmol/kg	6.9	20.7	69	6.1	18.3	61
$t_{1/2}$	hours	65	103	66.1	29.5	29.2	34.1
T_{max}	hours	18	16	20	4.5	5.25	5.5
C_{max}	nM	24.3	93.7	266.3	8.4	36.3	116.3

hProIFNa2b-Fc (dimer with two copies of IFN molecular):

1mg = 2 × weight (g) ÷ molecular weight (g/mol) = 2×10^{-3} (g) ÷ (144.5×10^{3}) (g/mol) = 13.84 nmol

Peg-IFN:

1mg = weight (g) ÷ molecular weight (g/mol) = 1×10^{-3} (g) ÷ (31×10^{3}) (g/mol) = 32.26 nmol

Supplementary Table 4. Acute toxicity study of hProIFNa2b-Fc in cynomolgus monkeys

Test article	Animal	Dose		Death	MTD*	
		mg/kg	nmol IFN/kg		mg/kg	nmol IFN/kg
hProIFNa2b-Fc (s.c.)	2 (1/sex)	20	276.82	0	60	830.45
	2 (1/sex)	40	553.63	0		
	2 (1/sex)	60	830.45	0		
Peg-IFN (s.c.)	4 (2/sex)	2.453	79.13	0	4.905	158.23
	4 (2/sex)	4.905	158.23	0		
	4 (2/sex)	9.81	316.45	2 (female)		

*MTD, Maximum Tolerated Dose.

Supplementary Table 5. Repeat-dose toxicity study of hProIFNa2b-Fc in rhesus monkeys

Test article	Animal	Dose		Death	NOAEL*	
		mg/kg	nmol IFN/kg		mg/kg	nmol IFN/kg
hProIFNa2b-Fc (s.c.)	10 (5/sex)	0.2	2.77	0	2	27.682
	10 (5/sex)	0.6	8.31	0		
	10 (5/sex)	2	27.7	0		
Peg-IFN (s.c.)	6 (3/sex)	0.118	3.81	0	0.118	3.81
	6 (3/sex)	0.3533	11.4	0		
	6 (3/sex)	1.18	38.1	1[†] (female)		

*NOAEL, No-Observed-Adverse-Effect Level.

[†]One female monkey was sacrificed moribund.

Supplementary Table 6. Reagent and resource

EAGENT or RESOURCE	SOURCE	IDENTIFIER
Antibodies		
InVivoMAb anti-mouse CD8 (53-5.8) (200 μg/mouse)	BioXcell	Cat# BE0223
Anti-CD45 (Flow cytometry, 30-F11) (1:500)	BioLegend	Cat# 103126
Anti-CD3 (Flow cytometry, 145-2C11) (1:400)	BD Biosciences	Cat# 564379
Anti-CD8 (Flow cytometry, 53-6.7) (1:500)	BioLegend	Cat# 100730
Anti-CD4 (Flow cytometry, RM4-5) (1:400)	BD Biosciences	Cat# 550954
Anti-CD11b (Flow cytometry, M1/70) (1:500)	BioLegend	Cat# 101236
Anti-CD11c (Flow cytometry, N418) (1:500)	BioLegend	Cat# 117306
Anti-MHCII (Flow cytometry, M5.114.15.2) (1:400)	eBioscience	Cat# 56-5321-82
Anti-F4/80 (Flow cytometry, REA126) (1:500)	Miltenyi Biotec	Cat# 130-102-422
Anti-Foxp3 (Flow cytometry, MF-14) (1:100)	BioLegend	Cat# 126408
Anti-Ki-67 (Flow cytometry, 16A8) (1:100)	BioLegend	Cat# 652404
Anti-PD-L1 (Flow cytometry, 10F.9G2) (1:500)	BioLegend	Cat# 124308
Anti-FcγIII/II receptor (clone 2.4G2) (1:100)	BD Biosciences	Cat# 553141
Goat anti-human IgG-HRP (1:1000)	Santa Cruz	sc-2453
Peroxidase AffiniPure Goat Anti-Human IgG (H+L) (1:2000)	Jackson ImmunoResearch	Cat# 109-035-088
AffiniPure Goat Anti-Human IgG, Fcγ fragment specific (1:650)	Jackson ImmunoResearch	Cat# 109-005-098
7-AAD Viability Staining Solution (Flow cytometry) (1:1000)	BioLegend	Cat# 420404
Chemicals, Peptides, and Recombinant Proteins		
TMB Solution (1X)	eBioscience	Cat# 00-4201-56
Dulbecco's Modified Eagle's Medium	Sigma- Aldrich	Cat# D6429
Collagenase type I	Sigma	Cat# C0130
DNase I	Roche	Cat# 11284932001
Critical Commercial Assays		
BD™ CBA Mouse Inflammation Kit	BD Biosciences	Cat# 552364
BD Mouse IFN-γ ELISPOT Sets	BD Biosciences	Cat# 551083
True-Nuclear™ Transcription Factor Buffer Set	BioLegend	Cat# 424401
EasySep Mouse CD11c Positive Selection Kit	STEMCELL	Cat# 18780
EasySep™ Mouse CD8+ T Cell Isolation Kit	STEMCELL	Cat# 19853
Experimental Models: Cell Lines		
B16	ATCC	Cat# CRL-6322
LLC	ATCC	Cat# CRL-1642™
MC38	ATCC	N/A
MDA-MB-231	ATCC	Cat# HTB-26™
FreeStyleTM 293-F	Thermo Fisher	Cat# R79007

Experimental Models: Organisms/Strains		
C57BL/6J	Jackson Laboratory	Cat# 000664
Cd11c-Cre	Jackson Laboratory	Cat# 008068
NSG-SGM3 mice	Jackson Laboratory	Cat# 013062
Ifnar1flox/flox mice	Dr. Ulrich Kalinke, Institute for Experimental Infection Research	N/A
Cd11c^{Cre}Ifnar1$^{flox/flox}$	This paper	N/A
Oligonucleotides		
Mouse MMP-2, forward primer 5’-CCAGCAAGTAGATGCTGCCT-3’	Sigma	N/A
Mouse MMP-2, reverse primer 5’-GATGGCATTCCAGGAGTCTG-3’	Sigma	N/A
Mouse MMP-9, forward primer 5’-TAGCTACCTCGAGGGCTTCC-3’	Sigma	N/A
Mouse MMP-9, reverse primer 5’-GTGGGACACATAGTGGGAGG-3’	Sigma	N/A
Mouse MMP-11, forward primer 5’-GCGAGGGGTACCTTCTGAG-3’	Sigma	N/A
Mouse MMP-11, reverse primer 5’-TAGGACCTTCACCTTCACGG-3’	Sigma	N/A
Mouse MMP-14, forward primer 5’-CCCAAGGCAGCAACTTCAG-3’	Sigma	N/A
Mouse MMP-14, reverse primer 5’-GTGAGCGTTGTGTGTGGGTA-3’	Sigma	N/A
Mouse FAP, forward primer 5’-TGCATTGTCTTACGTCCCTC-3’	Sigma	N/A
Mouse FAP, reverse primer 5’-TTGTTCTGAAATCCAGTTGGG-3’	Sigma	N/A
Mouse uPA, forward primer 5’-GCCCCACTACTATGGCTCTG-3’	Sigma	N/A
Mouse uPA, reverse primer 5’-ACAGATAAGCGGTCCTCCAG-3’	Sigma	N/A
Mouse TIMP-1, forward primer 5’-GTAAGGCCTGTAGCTGTGCC-3’	Sigma	N/A
Mouse TIMP-1, reverse primer 5’-AGGTGGTCTCGTTGATTCGT-3’	Sigma	N/A
Mouse TIMP-2, forward primer 5’-CGTTTTGCAATGCAGACGTA-3’	Sigma	N/A
Mouse TIMP-2, reverse primer 5’-GAATCCTCTTGATGGGGTTG-3’	Sigma	N/A
Mouse TIMP-3, forward primer 5’-CCAGGATGCCTTCTGCAAC-3’	Sigma	N/A
Mouse TIMP-3, reverse primer 5’-TAGACCAGAGTGCCAAAGGG-3’	Sigma	N/A

Mouse TIMP-4, forward primer 5'-ACACGCCATTTGACTCTTCC-3'	Sigma	N/A
Mouse TIMP-4, reverse primer 5'-CCCAGGGCTCAATGTAGTTG-3'	Sigma	N/A
Mouse 18s rRNA, forward primer 5'-GTAACCCGTTGAACCCCATT-3'	Sigma	N/A
Mouse 18s rRNA, reverse primer 5'-CCATCCAATCGGTAGTAGCG-3'	Sigma	N/A
Recombinant DNA		
Plasmid: pEE6.4-mIFNa4-Fc	This paper	N/A
Plasmid: pEE6.4-mProIFNa4-Fc	This paper	N/A
Plasmid: pEE6.4-hIFNa2b-Fc	This paper	N/A
Plasmid: pEE6.4-hProIFNa2b-Fc	This paper	N/A
Software and Algorithms		
GraphPad Prism software 7.0	GraphPad Software, Inc.	https://graphpad.com/scientific-software/prism/
CTL-ImmunoSpot® S6 Analyzer	Cellular Technology Limited	http://www.immunospot.com/ImmunoSpot-analyzers
CytExpert	Beckman Coulter, Inc	https://www.beckman.com/coulter-flow-cytometers/cytoflex/cytexpert
BD FACSChorus™ Software	BD Biosciences	https://www.bdbiosciences.com/en-us/instruments/research-instruments/research-software/flow-cytometry-acquisition/facschorus-software
FlowJo	Tree Star Inc.	https://www.flowjo.com/solutions/flowjo
Image Lab™ Software	Bio-Rad	http://www.bio-rad.com/en-us/category/image-analysis-software
