# Supplementary material for: Next generation of tumor-activating type I IFN enhances anti-tumor immune responses to overcome therapy resistance
Source: Nat Commun. 2021 Oct 7;12:5866. doi: 10.1038/s41467-021-26112-2 (PMC8497482; doi:10.1038/s41467-021-26112-2)
Supplement: Supplementary file 3 — Peer Review Fil [file 41467_2021_26112_MOESM3_ESM.pdf]

nature portfolio

Peer Review File

Next generation of tumor-activating type I IFN enhances antigen presentation to overcome treated-resistance inside tumor micro-environment

Open Access This file is licensed under a Creative Commons Attribution 4.0 International License, which permits use, sharing, adaptation, distribution and reproduction in any medium or format, as long as you give appropriate credit to the original author(s) and the source, provide a link to the Creative Commons license, and indicate if changes were made. In the cases where the authors are anonymous, such as is the case for the reports of anonymous peer reviewers, author attribution should be to 'Anonymous Referee' followed by a clear attribution to the source work. The images or other third party material in this file are included in the article's Creative Commons license, unless indicated otherwise in a credit line to the material. If material is not included in the article's Creative Commons license and your intended use is not permitted by statutory regulation or exceeds the permitted use, you will need to obtain permission directly from the copyright holder. To view a copy of this license, visit http://creativecommons.org/licenses/by/4.0/.

Editorial Note: This manuscript has been previously reviewed at another journal that is not operating a transparent peer review scheme. This document only contains reviewer comments and rebuttal letters for versions considered at *Nature Communications*. Mentions of prior referee reports have been redacted.

Reviewers' Comments:

Reviewer #1:
Remarks to the Author:
The authors have addressed previous comments.

Reviewer #2:
Remarks to the Author:
The authors have addressed the raised questions with a significant amount of new experiments. Being satisfied with their response and the enhanced quality of the manuscript, I do not have further questions.

Reviewer #5:
Remarks to the Author:
Cao et al designed a MMP responsive IFN-I, which was selectively activated in the tumor for enhanced safety but equivalent efficacy to the parental molecule. Although the similar prodrug design (masking with a MMP responsive linker) has been reported before, this strategy has been so far mostly applied in agonist antibodies. Therefore, the reported masked ProIFNa is novel. The characterizations and evaluation of ProIFNa were thorough. The studies were performed in several syngeneic mouse models, humanized mouse models, and monkeys. The added experiments (depletion, KO mice) in the revised manuscript strengthened the mechanistic understanding. The data interpretation was reasonable. Overall, this is a quite thorough piece of preclinical work. I would support the publication of this manuscript in Nature Communications.

There are several additional minor issues:

1. How was the overall survival of the syngeneic mouse models (Fig. 3)? Any complete responses? The author may want to show at least one example.
2. In general, the characterizations of ProIFNa were quite good. However, it is not clear how complete the masking IFNAR1/2 was removed. 100% or any residue receptor left? Tools such as Mass Spec may be used to collect such information.

Reviewer #6:
Remarks to the Author:
Regarding the "Preclinical studies of human ProIFN" section of the manuscript the following should be addressed:

1. In the results section presently titled "Preclinical Studies of human ProIFN", there is significant reporting of prior published studies associated with reference IFN molecules from "(Pharmacology Review, Intron A, 20-93)" – line 259; and from "Toxicologists' Review. BLA 99-1488" – lines 263 and 272. Discussion of the findings from these prior published reports and comparison (both PK and tolerability) to the findings reported in this study should be placed in the Discussion. The Results section should be limited to the results coming from the novel work being reported in the manuscript. Furthermore, the reference documents being discussed should be cited in the bibliography in a standard format (rather than how they are now in the text) to enable readers to

access and review. This would also pertain to reference/discussion of the human NOAEL that is reported/cited on line 281 (which should also have a citation unless it is being reported here as result – in which case more details should be provided on how/where that number was deduced)

2. The Results subtitle “Preclinical studies of human ProIFN” is somewhat vague – it should be reflective of the subject topic and the significance of the data in that section – would suggest something like “ProIFN pharmacokinetics and tolerability in non-human primates”

3. For the mouse efficacy studies – the dose for ProIFN molecule is reported in nmol – while the dose for the NHP studies is in mg/kg. To enable cross comparison of the efficacy data with the safety data – the dose of ProIFN administered in the mouse studies should also be provided in mg/kg – at least once in the manuscript.

4. Regarding the stability studies there is a comment (line 255) that the molecule did not lose activity. However, from what I can deduce from Supplementary Table 1, there are no activity assays such as a potency assay performed – rather these are more measures of biophysical intactness. Ideally inclusion of some measure of a maintained level of bioactivity during prolonged storage should be included.

REVIEWERS' COMMENTS

Reviewer #1 (Remarks to the Author):

The authors have addressed previous comments.

Reviewer #2 (Remarks to the Author):

The authors have addressed the raised questions with a significant amount of new experiments. Being satisfied with their response and the enhanced quality of the manuscript, I do not have further questions.

Reviewer #5 (Remarks to the Author): to replace original Reviewers #3-4

Cao et al designed a MMP responsive IFN-I, which was selectively activated in the tumor for enhanced safety but equivalent efficacy to the parental molecule. Although the similar prodrug design (masking with a MMP responsive linker) has been reported before, this strategy has been so far mostly applied in agonist antibodies. Therefore, the reported masked ProIFNa is novel. The characterizations and evaluation of ProIFNa were thorough. The studies were performed in several syngeneic mouse models, humanized mouse models, and monkeys. The added experiments (depletion, KO mice) in the revised manuscript strengthened the mechanistic understanding. The data interpretation was reasonable. Overall, this is a quite thorough piece of preclinical work. I would support the publication of this manuscript in Nature Communications.

There are several additional minor issues:

1. How was the overall survival of the syngeneic mouse models (Fig. 3)? Any complete responses? The author may want to show at least one example.

We thank the reviewer for the suggestion. We have included cured tumors percentage in the LLC tumor model (Fig. R1). We have updated Fig. R1 in Fig. 3E in the manuscript.

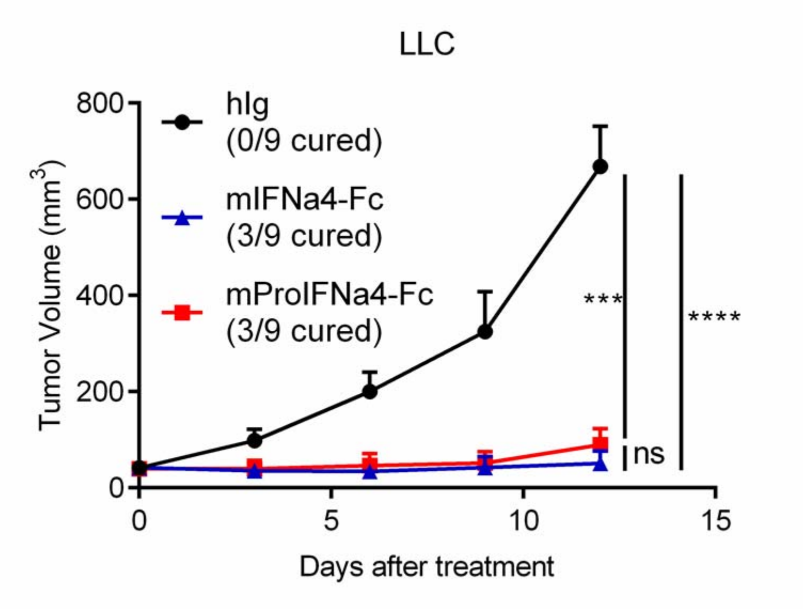

Figure R1. Masked IFN-I shows tumor-targeting and reduced toxicity. C57BL/6J mice were s.c. inoculated with $1x10^6$ LLC tumor cells and i.p. treated with 1 nmol of hIg, mIFNa4-Fc, or mProIFNa4-Fc, on day 12, 15, and 18 (n=9). Tumor growth was shown.

2. In general, the characterizations of ProIFNa were quite good. However, it is not clear how complete the masking IFNAR1/2 was removed. 100% or any residue receptor left? Tools such as Mass Spec may be used to collect such information.

We thank the reviewer for the question. We designed the ProIFN as IFNAR2-Linker-IFNa-Fc. The sequence of the linker G_4S-**Sbustrate**-G_4S was GGGGS**SGQLLGFLTA**GGGGS. The cutting site for the enzyme was inside the substrate. After cleavage by enzyme, the whole IFNAR2 should be removed without any residue receptor left. Please check Fig. R2 (Fig. 1E in the manuscript), the Non-sub control without the substrate cannot be cleaved by enzyme, suggesting there was no additional cutting site inside the masking receptor. Therefore, the only cutting sites was inside the substrate and the whole receptor will be removed after cleavage.

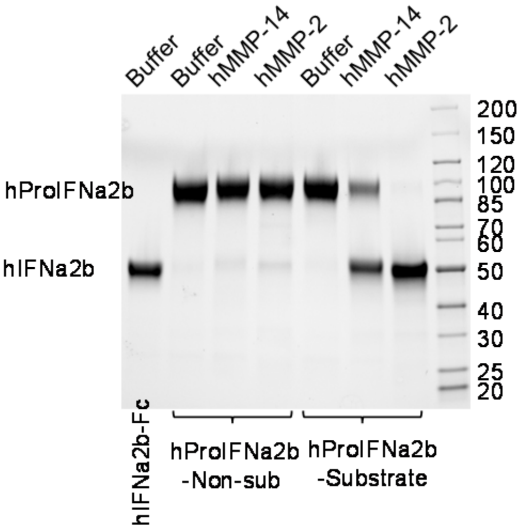

Figure R2. Reducing SDS-PAGE analysis of hProIFNa2b-Fc as well as MMP-14- or MMP-2-cleaved hProIFNa2b-Fc.

Reviewer #6 (Remarks to the Author):

Regarding the “Preclinical studies of human ProIFN” section of the manuscript the following should be addressed:

1. In the results section presently titled “Preclinical Studies of human ProIFN”, there is significant reporting of prior published studies associated with reference IFN molecules from “(Pharmacology Review, Intron A, 20-93)” – line 259; and from “Toxicologists’ Review. BLA 99-1488” – lines 263 and 272. Discussion of the findings from these prior published reports and comparison (both PK and tolerability) to the findings reported in this study should be placed in the Discussion. The Results section should be limited to the results coming from the novel work being reported in the manuscript. Furthermore, the reference documents being discussed should be cited in the bibliography in a standard format (rather than how they are now in the text) to enable readers to access and review. This would also pertain to reference/discussion of the human NOAEL that is reported/cited on line 281 (which should also have a citation unless it is being reported here as result –

in which case more details should be provided on how/where that number was deduced)

We thank the reviewer for the suggestion and question. We have moved all the prior published reports and comparison to the Discussion. The two reference documents are cited in the bibliography in a standard format now. How the numbers were deduced from mg to nmol in Supplementary Table 3-5 were shown as following:

hProIFNa2b-Fc (dimer with two copies of IFN molecular):

1mg = 2 × weight (g) ÷ molecular weight (g/mol) = 2×10-3 (g) ÷ (144.5×103) (g/mol) = 13.84 nmol

Peg-IFN:

1mg = weight (g) ÷ molecular weight (g/mol) = 1×10-3 (g) ÷ (31×103) (g/mol) = 32.26 nmol

We have included the information under Supplementary Table 3 in the manuscript.

2. The Results subtitle “Preclinical studies of human ProIFN” is somewhat vague – it should be reflective of the subject topic and the significance of the data in that section – would suggest something like “ProIFN pharmacokinetics and tolerability in non-human primates”

We thank the reviewer for the suggestion. The subtitle “Preclinical studies of human ProIFN” is replaced with “ProIFN pharmacokinetics and tolerability in non-human primates”.

3. For the mouse efficacy studies – the dose for ProIFN molecule is reported in nmol – while the dose for the NHP studies is in mg/kg. To enable cross comparison of the efficacy data with the safety data – the dose of ProIFN administered in the mouse studies should also be provided in mg/kg – at least once in the manuscript.

We thank the reviewer for the question. We have included the description of mg/kg in the figure legend of Fig. 6 (A-B). In this experiment, the dose of 1 nmol of mProIFNa4-Fc is about 8 mg/kg for the mouse.

4. Regarding the stability studies there is a comment (line 255) that the molecule did not lose activity. However, from what I can deduce from Supplementary Table 1, there are no activity assays such as a potency assay performed – rather these are more measures of biophysical intactness. Ideally inclusion of some measure of a maintained level of bioactivity during prolonged storage should be included.

We thank the reviewer for the question. In Supplementary Table 2 about the stability study of hProIFNa2b-Fc, the bioactivity of prolonged stored ProIFN was measured by the IFN-I activity reporter assay. We are sorry for the unclear description and have included in the main text (line 255, highlight in Yellow).
